# Common mechanisms of physiological and pathological rupture events in biology: novel insights into mammalian ovulation and beyond

**DOI:** 10.1111/brv.12970

**Published:** 2023-05-08

**Authors:** Emily J. Zaniker, Elnur Babayev, Francesca E. Duncan

**Affiliations:** Department of Obstetrics and Gynecology, Feinberg School of Medicine, Northwestern University, 303 E. Superior Street, Lurie 10-109, Chicago, IL 60611, USA

**Keywords:** ovulation, rupture, aneurysm, chorioamniotic membrane, follicle, parturition

## Abstract

Ovulation is a cyclical biological rupture event fundamental to fertilization and endocrine function. During this process, the somatic support cells that surround the germ cell undergo a remodelling process that culminates in breakdown of the follicle wall and release of a mature egg. Ovulation is driven by known proteolytic and inflammatory pathways as well as structural alterations to the follicle vasculature and the fluid-filled antral cavity. Ovulation is one of several types of systematic remodelling that occur in the human body that can be described as rupture. Although ovulation is a physiological form of rupture, other types of rupture occur in the human body which can be pathological, physiological, or both. In this review, we use intracranial aneurysms and chorioamniotic membrane rupture as examples of rupture events that are pathological or both pathological and physiological, respectively, and compare these to the rupture process central to ovulation. Specifically, we compared existing transcriptomic profiles, immune cell functions, vascular modifications, and biomechanical forces to identify common processes that are conserved between rupture events. In our transcriptomic analysis, we found 12 differentially expressed genes in common among two different ovulation data sets and one intracranial aneurysm data set. We also found three genes that were differentially expressed in common for both ovulation data sets and one chorioamniotic membrane rupture data set. Combining analysis of all three data sets identified two genes (*Angptl4* and *Pfkfb4*) that were upregulated across rupture systems. Some of the identified genes, such as *Rgs2*, *Adam8*, and *Lox*, have been characterized in multiple rupture contexts, including ovulation. Others, such as *Glul*, *Baz1a*, and *Ddx3x*, have not yet been characterized in the context of ovulation and warrant further investigation as potential novel regulators. We also identified overlapping functions of mast cells, macrophages, and T cells in the process of rupture. Each of these rupture systems share local vasoconstriction around the rupture site, smooth muscle contractions away from the site of rupture, and fluid shear forces that initially increase and then decrease to predispose one specific region to rupture. Experimental techniques developed to study these structural and biomechanical changes that underlie rupture, such as patient-derived microfluidic models and spatiotemporal transcriptomic analyses, have not yet been comprehensively translated to the study of ovulation. Review of the existing knowledge, transcriptomic data, and experimental techniques from studies of rupture in other biological systems yields a better understanding of the physiology of ovulation and identifies avenues for novel studies of ovulation with techniques and targets from the study of vascular biology and parturition.

## I. INTRODUCTION

The process of ovulation is a highly spatiotemporally coordinated event that culminates in rupture of the wall of the ovarian follicle, which consists of somatic support cells, and expulsion of the gamete and its surrounding specialized cumulus cells (Duffy *et al*., 2019; Richards, 2005; Richards *et al*., 1998; Russell & Robker, 2007). The rupture event itself takes place over the course of several hours after the hormonal trigger of ovulation, but the formation of a site within the follicle predisposed to rupture occurs earlier in folliculogenesis (Duffy *et al*., 2019; Espey, 1967; Martin & Talbot, 1987; Richards *et al*., 1998). The process of follicle rupture is characterized by extracellular matrix (ECM) reorganization, inflammation, and structural changes that occur in a distinct spatiotemporal pattern, a process that is relatively unique in the context of normal physiological processes in the body (Brown *et al*., 2010; Duffy *et al*., 2019; Lind *et al*., 2006; Park *et al*., 2020; Richards, Liu & Shimada, 2008). In addition to ovulation, multiple other types of rupture that mirror some of the features of ovulation exist in humans. Many of these ruptures are pathological, such as rupture of intracranial aneurysms (ICAs). Rupture of the chorioamniotic membrane at the time of parturition can be either physiological or pathological depending on the timing of the rupture. Despite the clear differences between the circumstances of ovulation, aneurysms, and chorioamniotic membrane rupture, there are broad similarities relating to structural remodelling and inflammation that link these processes. In this review, we use ovulation, ICAs, and chorioamniotic membrane rupture as examples of rupture systems that are respectively physiological, pathological, or both, to inform common mechanisms.

Although similarities exist between these examples on a pathophysiological level, there are no current studies that compare transcriptomic pathways that lead to rupture across these systems. The process of rupture in ICAs and chorioamniotic membranes has been well characterized because the consequences of rupture in these systems can be life threatening (Lindekleiv *et al*., 2015; Siegler, Weiner & Solt, 2020). There have been relatively few transcriptomic studies on ovulation and even fewer assessing what makes a follicle rupture or fail to rupture at a spatiotemporal level. In this review, we use data identified in the context of ICAs and chorioamniotic membranes and compare it with the limited data sets available for ovulation to identify pathways that are central to the concept of rupture and remodelling. Through this strategy we identify potential gene targets for future mechanistic studies and drug discovery and develop a new framework for the study of the spatiotemporal transcriptomic changes that underlie rupture.

In addition to the spatiotemporal transcriptomic changes that underlie rupture in each of these systems, there is evidence of structural and biomechanical changes that predispose rupture to occur. These changes include spatial alterations in pressure and fluid shear forces, vasoconstriction of microvasculature, and contraction of smooth muscle cells (Cavender & Murdoch, 1988; Cebral *et al*., 2011; Chowdhury *et al*., 2014; Martin & Talbot, 1981*b*; Matousek *et al*., 2001; Shojima *et al*., 2004). In this review, we identify where parallels exist that may provide a greater understanding of the basic biology of each condition, particularly in ovulation where few modern studies on biomechanical and structural changes associated with follicle rupture have been conducted. Additional discoveries about follicle rupture derived from this review could also aid in the understanding of ovarian conditions associated with altered follicle rupture, such as luteinized unruptured follicle syndrome or reproductive aging. These comparisons do not mean that these rupture processes are identical or that all of the identified parallels are exact. However, we argue that new insights can be drawn and new experiments can be designed when commonalities are explored, and knowledge is shared across these distinct fields.

## II. OVERVIEW OF RUPTURE SYSTEMS

### (1) Overview of ovulation and follicle rupture

**Fig. 1.**
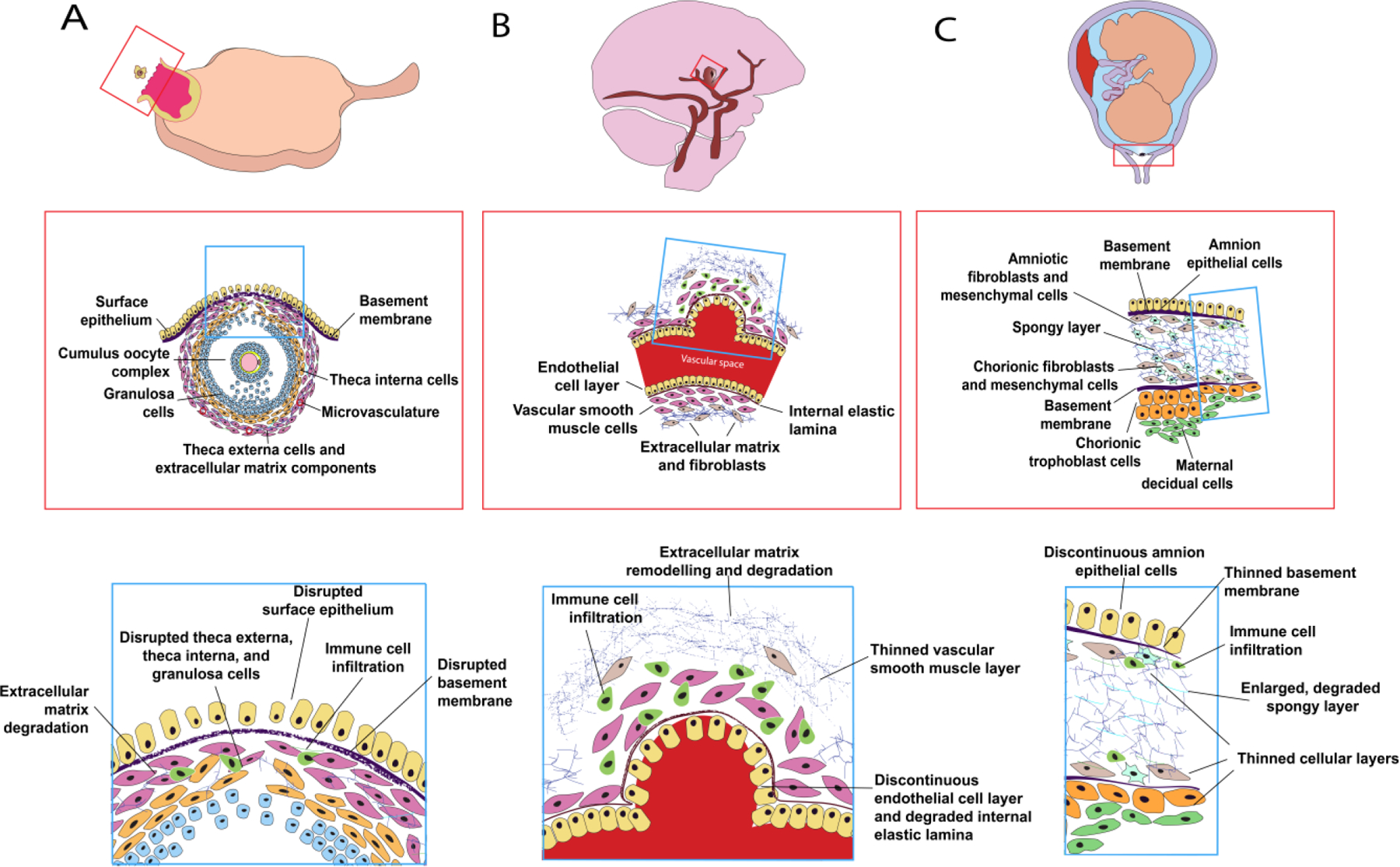
Comparison of rupture systems for (A) ovulation, (B) intracranial aneurysms, and (C) chorioamniotic membranes. The orange box identifies the region of interest. The blue box indicates the specific zone with rupture potential. These rupture events can be described as physiological (A), pathological (B), or both (C). All three types of rupture consist of similar remodelling processes, including disrupted cellular layers, remodelling of the extracellular matrix, and immune cell infiltration.

The follicle is the functional unit of the ovary and consists of an oocyte surrounded by somatic support cells known as the granulosa and theca cells (Rodgers & Irving-Rodgers, 2010; Russell & Robker, 2007; Smith *et al*., 2011). During the process of oogenesis and folliculogenesis, the germ cell and somatic cells grow in a coordinated fashion ultimately to form a fully differentiated pre-ovulatory antral follicle (DiZerega & Hodgen, 1981; Richards *et al*., 1998; Rimon-Dahari *et al*., 2016; Rodgers & Irving-Rodgers, 2010). The follicle wall is composed of granulosa cells that support oocyte development and hormone production, a basement membrane enriched in collagen, and layers of theca cells that facilitate androgen synthesis (Fig. 1A) (Lind *et al*., 2006; Richards *et al*., 1998; Smith *et al*., 2011). As the follicle grows and matures, the support cells multiply and follicular fluid is pumped into the follicle as a result of angiogenesis and contractile activity of smooth muscle-like cells (Duffy *et al*., 2019; Espey, 1967; Martin & Talbot, 1981*a*, 1987; Talbot & Chacon, 1982). This period of follicle and oocyte development marks the follicular phase of the menstrual cycle and lasts for approximately 14 days in humans (Richards *et al*., 1998). At the end of this phase in the menstrual cycle, there is a surge of luteinizing hormone (LH) which induces ECM degradation, disruption of the basement membrane and ovarian surface epithelium, disruption and thinning of somatic cell layers, and immune cell infiltration, all of which facilitate ovulation (Fig. 1A) (Direito *et al*., 2013; Duffy *et al*., 2019; Espey, 1967; Lind *et al*., 2006; Park *et al*., 2020; Richards *et al*., 2008, 1998; Russell & Robker, 2007). Ovulation is complete when a cumulus oocyte complex (COC) is released from the ovulatory follicle at the surface of the ovary (Richards *et al*., 1998). The COC initially remains attached to the surface of the ovary and is gradually transported through the oviduct by ciliated cells (Bylander *et al*., 2015; Croxatto, 2002; Kölle *et al*., 2009; Lyons, Saridogan & Djahanbakhch, 2006; Talbot, Geiske & Knoll, 1999). After ovulation, the unruptured parts of the follicle involute to form the corpus luteum, a temporary endocrine structure that produces oestrogen and progesterone to support early pregnancy (Niswender *et al*., 2000; Plant, Zeleznik & Albertini, 2015; Richards *et al*., 1998; Stocco, Telleria & Gibori, 2007). The average woman will undergo ovulation nearly 400 times during her lifetime, averaging around 12 ovulations per year for around 40 fertile years (Clow, Hurst & Fleming, 2002). This number may be lower depending on pregnancies, ovarian pathologies, and use of contraceptive medications that block ovulation.

Although the structural changes associated with follicle rupture and ovulation are most distinct after the LH surge, structural asymmetry develops prior to this hormone surge during the follicular phase. This asymmetry can be seen during the formation of the antral cavity (DiZerega & Hodgen, 1981; Rimon-Dahari *et al*., 2016). Prior to ovulation, one side of the follicle, the future site of rupture, begins to widen and swell as the layer of cells thins, while the other side, the future site of corpus luteum formation, remains compact with a thicker cellular layer (Duffy *et al*., 2019). Preliminary work to understand the mechanism of the asymmetry that underlies follicular development and rupture has largely focused on the temporal changes that contribute to follicular growth and remodelling. The asymmetry appears to be driven by inflammatory mediators, ECM remodelling, and angiogenic changes that regulate antral fluid flow (Duffy *et al*., 2019). The consistent pattern of these asymmetrical changes implies that there is a functionally and molecularly distinct sidedness to the ovulatory follicle prior to rupture.

The combination of inflammatory signalling, fluid pressure, and ECM remodelling that occurs in a spatiotemporal pattern in follicle rupture mirrors the changes that occur in other well-characterized pathological and physiological rupture events in the human body. Understanding follicle rupture on a spatiotemporal level could provide insight into conditions including luteinized unruptured follicle syndrome and ovarian aging which involve impaired follicle rupture and subfertility (Mara *et al*., 2020; Marik & Hulka, 1978). It may also uncover targets for development of novel non-hormonal contraceptives that inhibit follicle rupture without interfering with hormone production and luteinization.

### (2) Overview of intracranial aneurysm formation and rupture

ICAs are structurally diseased regions of neurovascular tissue that form when, due to a variety of complex factors, a section of an intracranial blood vessel stretches, and the vessel wall weakens (Keedy, 2006). The most common type of ICA is a saccular aneurysm, which forms due to breakdown of internal layers of the blood vessel wall (Keedy, 2006). Saccular aneurysms most commonly form in a specific region of the brain arterial system termed the Circle of Willis (Keedy, 2006). Arteries within the Circle of Willis consist of an inner layer of endothelial cells, a layer of elastic tissue called the internal elastic lamina, a layer of vascular smooth muscle cells, and an ECM layer with fibroblasts (Fig. 1B) (Savastano *et al*., 2018). When an aneurysm forms, the structure of the vasculature changes due to ECM remodelling and degradation, degraded internal elastic lamina, disrupted endothelial layer, thinned smooth muscle layer, and immune cell infiltration (Fig. 1B) (Keedy, 2006; Savastano *et al*., 2018). Aneurysms may be asymptomatic until the point at which they rupture. When an ICA within the Circle of Willis ruptures, it causes a subarachnoid haemorrhage which can trigger a haemorrhagic stroke (Savastano *et al*., 2018). It is difficult to estimate the frequency of unruptured ICAs accurately as they are often asymptomatic and only discovered incidentally. Broad estimates suggest that 1–5% of the population may have an ICA and the incidence of subarachnoid haemorrhages, frequently caused by ruptured ICA, was reported as 7.9 per 100,000 person-years, a measurement of the incidence rate relative to the duration of a study, in one large study (Etminan *et al*., 2019; Wiebers, 2003). Subarachnoid haemorrhages due to ruptured aneurysm cause around 5% of all reported strokes (Nieuwkamp *et al*., 2009). The rate at which ICA rupture occurs varies widely but is estimated to be around 1.6% annually (Korja, Lehto & Juvela, 2014). Although ruptured aneurysms pose a higher morbidity and mortality rate than those that do not rupture, the one-year mortality rate for patients with unruptured aneurysms was found in one study to be 2.7% compared to 15.6% for patients with ruptured aneurysms (Lindekleiv *et al*., 2015). Mechanistic studies of ICA suggest that a combination of inflammatory signalling, fluid-derived shear stress on arterial walls, and ECM remodelling cause structural changes in the blood vessel walls that lead to ICA formation (Kanematsu *et al*., 2011; Kurki *et al*., 2011; Medero *et al*., 2020; Savastano *et al*., 2018; Shojima *et al*., 2004). This remodelling often occurs asymmetrically and creates a particular region of weakness in the vessel wall that makes it susceptible to rupture (Nehls *et al*., 1985; Zhang *et al*., 2016). Understanding the mechanisms of ICA rupture could lead to the development of treatment approaches that target the likely rupture zone of the aneurysm and reduce the risk of rupture in patients with identified ICAs.

### (3) Overview of physiological and pathological chorioamniotic membrane rupture

The chorioamniotic membrane forms early in fetal development when two membranes, the chorion and the amnion, fuse at 12 weeks gestation (Bourne, 1962; Bryant-Greenwood, 1998). The chorion (trophoblast layer, basement membrane, fibroblasts, and mesenchymal cells) forms the outermost layer of the amniotic sac and the amnion (spongy layer, fibroblasts, mesenchymal cells, basement membrane, and epithelial cells) forms the inner lining of the sac that fills with amniotic fluid and cushions the growing fetus (Fig. 1C) (Bourne, 1962; Bryant-Greenwood, 1998; Kumar *et al*., 2016). Rupture of the chorioamniotic membrane occurs as a result of epithelial cell disruption, immune infiltration, thinning of basement membrane and cellular layers, and degradation of the spongy layer components (Fig. 1C) (Kumar *et al*., 2016; Menon & Richardson, 2017). In a full-term pregnancy, rupture of the chorioamniotic membrane occurs at the onset of labour (Plant *et al*., 2015). This rupture can also happen pathologically if it occurs prior to labour. Premature rupture of membranes (PROM) is a condition where the chorioamniotic membrane ruptures after 37 weeks gestation but prior to the onset of labour (Menon & Richardson, 2017). Preterm premature rupture of membranes (PPROM) is a condition where the chorioamniotic membrane ruptures before 37 weeks gestation (Menon & Richardson, 2017). PROM occurs in an estimated 8% of all pregnancies in the USA and PPROM occurs in 2–3% of all pregnancies in the USA (Siegler *et al*., 2020). Both PROM and PPROM carry risk of intrauterine infection that can cause serious complications for the mother (Siegler *et al*., 2020). PPROM can also be accompanied by abruptio placentae, where the placenta detaches from the uterine wall (Major *et al*., 1995). PPROM also carries serious risks for the fetus, including respiratory distress, haemorrhage, impaired neurodevelopment, and fetal demise (Siegler *et al*., 2020). In around 1% of cases, PPROM can occur prior to 22 weeks gestation when the fetus is pre-viable (Bendix *et al*., 2015; Siegler *et al*., 2020; Waters & Mercer, 2009). This carries a much higher risk of fetal demise, estimated at 57.7% of cases prior to 22 weeks compared to 14.4% of cases between 22 and 37 weeks (Siegler *et al*., 2020). Membrane rupture, both pathological and physiological, is associated with inflammatory pathways, ECM remodelling, and mechanical forces from smooth muscle contractions (Bryant-Greenwood, 1998; Joyce, Moore & Sacks, 2009; Kumar *et al*., 2016). There is evidence that pathological rupture of the chorioamniotic membrane is associated with a pro-inflammatory state and accelerated remodelling (Gomez-Lopez *et al*., 2011; Kumar *et al*., 2016; Moore *et al*., 2009). The rupture zone is also spatially organized with rupture occurring most often at the region of the membrane over the cervix (Kumar *et al*., 2016; Lappas *et al*., 2008; McLaren, Taylor & Bell, 1999). Understanding the spatiotemporal dynamics of chorioamniotic membrane rupture could lead to the development of rupture prediction tools and treatments to prevent premature rupture of membranes.

## III. TRANSCRIPTOMIC DATA SET COMPARISON FOR DIFFERENT RUPTURE SYSTEMS

To assess common pathways between non-ovulation rupture systems and ovulation, we first surveyed the literature to find appropriate data sets for comparison. Several molecular pathways have been implicated in assessment of rupture risk of ICAs. While many of these pathways have been identified by examining expression patterns of individual genes within aneurysmal tissues, some studies have attempted to assess expression in a more comprehensive manner. Oligonucleotide microarrays have been used to compare expression of transcripts of ruptured and unruptured ICAs to control tissues and found no significant differences (Krischek *et al*., 2008; Shi *et al*., 2009). Studies with slightly higher sample sizes found less than 20 differentially expressed genes (DEGs) between samples (Marchese *et al*., 2010; Pera *et al*., 2010). By contrast, another study identified over 1,400 DEGs between ruptured and unruptured ICA walls (Kurki *et al*., 2011) (Table 1). This difference is likely due to the larger sample size and updated microarray probes used by Kurki *et al*. (2011). This data set provides a wealth of information that can be used to identify pathways that contribute to the process of rupture.

The data sets investigating chorioamniotic membrane rupture vary because chorioamniotic membrane rupture can be both physiological and pathological. Many comprehensive transcriptomic data sets relating to chorioamniotic membrane rupture focus on pathological rupture because of a compelling clinical interest in developing screening methods and treatments for PROM and PPROM. A comparison of chorioamnion samples from four term and four preterm deliveries identified 252 upregulated and 18 downregulated genes in the preterm group (Pereyra *et al*., 2019). Another study characterized differential microRNA (miRNA) expression in patients at term without labour, at term with labour, and preterm with labour using microarray analysis (Montenegro *et al*., 2009). They identified dozens of miRNAs that may be involved in post-transcriptional regulation of genes leading to chorioamniotic membrane rupture. One study focused on spontaneous rupture of membranes at term and assessed regional differences in the chorioamniotic membranes at the site over the cervix that ruptures and at sites distal to the rupture zone (Nhan-Chang *et al*., 2010) (Table 1). They identified 677 DEGs between ruptured and non-ruptured sites in their microarray analysis and selected several genes for validation using quantitative reverse transcription polymerase chain reaction (qRT-PCR) (Nhan-Chang *et al*., 2010). This data set is well suited for comparison to other rupture systems as it investigated the mechanism of spatial differences in rupture within a physiological rupture system.

Although there have yet to be any comprehensive analyses of the transcriptomic changes that differentiate the specific ruptured zone on the apical side of the follicle from the unruptured zone in the context of ovulation, there are several studies that have interrogated transcriptomic changes associated with the general rupture process. One study compared ovulating follicles to an anovulatory model induced by knockout of the nuclear progestin receptor (*Pgr*) in a zebrafish (*Danio rerio*) model (Liu *et al*., 2017) (Table 1). This study found 2,888 DEGs between the ruptured and unruptured follicles. A *Pgr*-knockout model was also used in the mouse to interrogate the anovulatory phenotype. One study performed bulk RNA-sequencing on granulosa cells isolated from hormonally stimulated mouse ovaries at 10 h post-injection of human chorionic gonadotropin (hCG) (Smith *et al*., 2022), and another study performed single-cell RNA-sequencing on dissociated whole ovaries at 6 h post-hCG injection (Park *et al*., 2020). The latter study used markers specific to ovulatory mural granulosa cells to isolate this population for analysis of DEGs between wildtype mice and anovulatory *ESR2-Pgr*KO mice, making this study particularly suitable for comparison to other ruptured tissues due to the demonstrated purity of the mural granulosa cell population (Table 1). Other knockouts of genes essential for ovulation, including endothelin-2 and the heterodimeric transcription factors known as core binding factors, are either not fully anovulatory or cause alterations to other key ovulatory functions such as luteinization (Cacioppo *et al*., 2017; Lee-Thacker *et al*., 2020). For the purposes of this review, we compared the data sets of Liu *et al*. (2017) and Park *et al*. (2020) for ovulation, with those of Kurki *et al*. (2011) for ICAs and Nhan-Chang *et al*. (2010) for chorioamniotic membrane rupture as these studies were designed explicitly to compare ruptured *versus* unruptured tissue (Table 1). Note that the ovulation data sets represent data from zebrafish and mice. Since suitable data from human ovulatory follicles are not yet available, the use of two different species for comparisons with human tissues allows more robust conclusions. Park *et al*. (2020) presented their data as positive values showing upregulated genes in unruptured tissue, whereas data for the other three studies were presented with positive values indicating upregulation in ruptured tissue. We therefore transformed the values from Park *et al*. (2020) to reflect the same convention used in the other studies, for ease of comparison.

### (1) Common transcriptomic changes across rupture systems

**Fig. 2.**
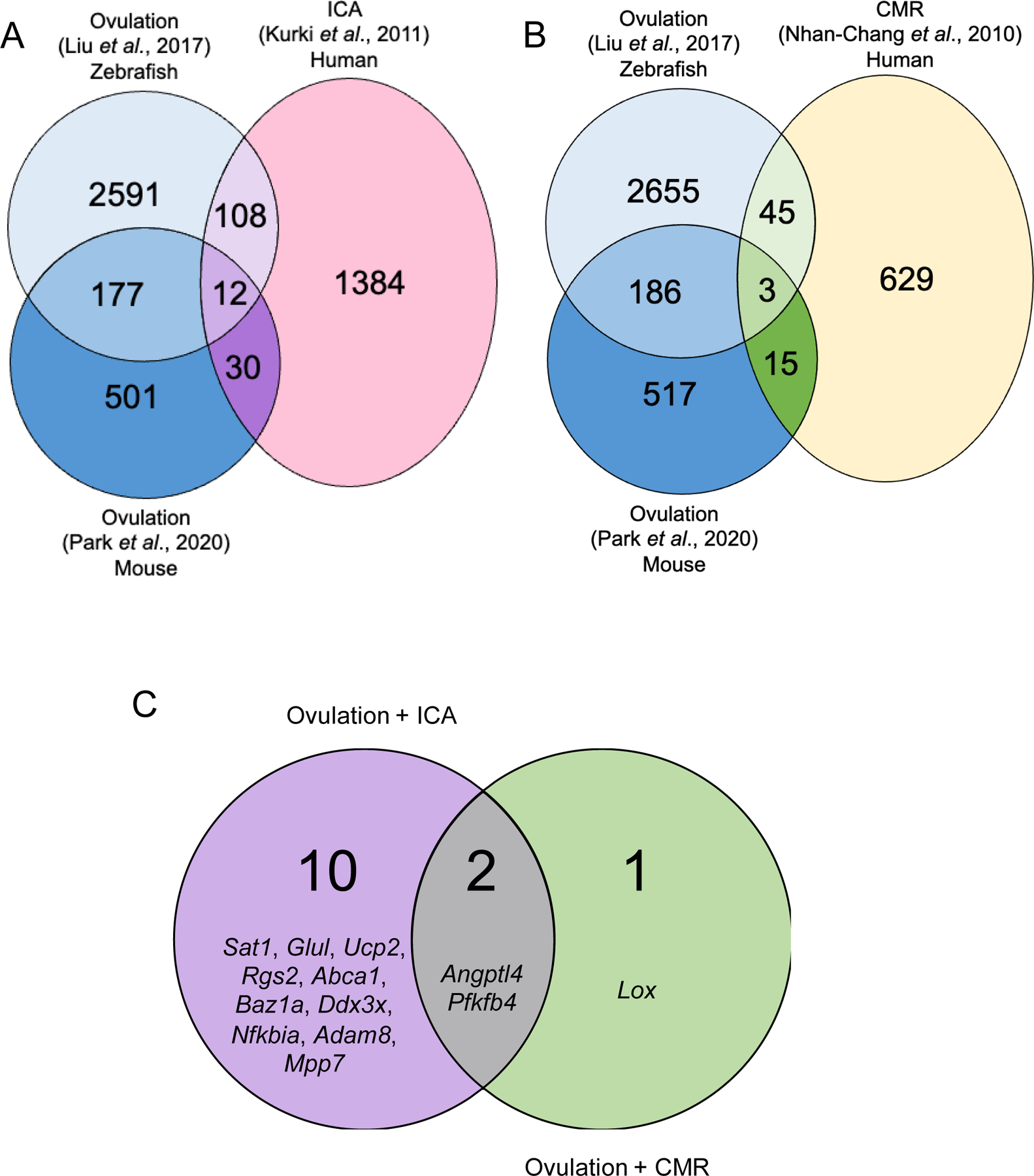
Comparison of the selected data sets for different rupture systems: Kurki *et al*. (2011) for intracranial aneurysm (ICA); Nhan-Chang *et al*. (2010) for chorioamniotic membrane rupture (CMR); and both Liu *et al*. (2017) and Park *et al*. (2020) for ovulation. Data sets were compared to identify overlapping genes, and these lists were filtered for genes with changes in expression in the same direction (i.e. both upregulated or both downregulated). The Venn diagrams show which genes were in common for the comparison of ovulation and ICA (A) and the comparison between ovulation and CMR (B). Two genes (*Angptl4*, and *Pfkfb4*) were upregulated in all data sets (C).

Using the selected data sets, comparisons were made between the reported DEGs to identify pathways that may be common between rupture processes (Fig. 2A, B). A comparison of the transcriptional changes reported in Kurki *et al*. (2011) and Liu *et al*. (2017) revealed 120 genes (68 upregulated, 52 downregulated) that were differentially expressed in the same direction in both studies (Fig. 2A). A comparison between Kurki *et al*. (2011) and Park *et al*. (2020) revealed 42 genes (32 upregulated, 10 downregulated) that were differentially expressed in the same direction in both studies (Fig. 2A). From these three studies, there was a total of 12 overlapping genes (Fig. 2A; Table 2; see online supporting information, Table S1). A comparison between Nhan-Chang *et al*. (2010) and Liu *et al*. (2017) found 48 genes (30 upregulated, 18 downregulated) that were differentially expressed in the same direction in both studies (Fig. 2B). A comparison between Nhan-Chang *et al*. (2010) and Park *et al*. (2020) found 18 genes (10 upregulated, 8 downregulated) that were differentially expressed in the same direction in both studies (Fig. 2B). When the lists of genes generated from the chorioamniotic membrane rupture–ovulation analyses were compared, there was a total of three overlapping genes (Fig. 2B; Table 3; Table S2). There were two genes that overlapped across all comparisons (Fig. 2C). Although not a focus of this review, there were 60 genes in common between Kurki *et al*. (2011) and Nhan-Chang *et al*. (2010) that did not overlap with either ovulation data set; these genes may elucidate processes related to rupture that are distinct from those involved in ovulation (Table S3).

Although the majority of these genes in common between ovulation and other rupture systems are unique to each comparison, two transcripts were present in all comparisons (Fig. 2C). Angiopoietin like 4 (*Angptl4*) is an angiogenic factor and lipid metabolism regulator that has been characterized in cumulus cells and in cases of polycystic ovarian syndrome (PCOS) (Al-Edani *et al*., 2014; Chaemsaithong *et al*., 2013; Haddad *et al*., 2006; Jiang *et al*., 2022; Kremer *et al*., 2016; Nhan-Chang *et al*., 2010). It is also associated with arteriovenous malformations in the brain that can lead to ICAs and is present in multiple transcriptomic studies of aneurysm rupture (Estrelinha, Hinterseher & Kuivaniemi, 2014; Gäbel *et al*., 2017; Mikhak *et al*., 2011; Xu *et al*., 2017). It is also upregulated in the cervix and myometrium during labour and may initiate labour within the myometrium (Bollopragada *et al*., 2009; Mittal *et al*., 2010; Weiner *et al*., 2010). 6-phosphofructo-2-kinase/fructose-2,6-biphosphatase 4 (*Pfkfb4*) is involved in stress and hypoxia responses within cells (Minchenko *et al*., 2004). *Pfkfb4* has been characterized in multiple transcriptomic studies from peri-ovulatory granulosa cells and is upregulated in cumulus cells following hCG stimulation (Cruz *et al*., 2017; Poulsen *et al*., 2020). It is also enriched during subarachnoid haemorrhage following ICAs (Yan *et al*., 2021). *Pfkfb4* is also known to participate in initiating myometrial contractions during term labour (Weiner *et al*., 2010).

Although the list of genes in common between multiple rupture data sets and ovulation is small, the observed overlap provides insight into pathways and specific transcripts that are enriched in all these rupture systems and suggests avenues for deeper investigation. Assessing both the unique features of each rupture system and the similarities to ovulation, both from a transcriptomic level and a functional or structural level, will provide additional insights into the biology of all these rupture systems.

There are some limitations to our analysis. For example, the data sets compared here are not perfectly analogous in how they assessed rupture or rupture potential. Kurki *et al*. (2011) compared ruptured and unruptured ICA tissue from different individuals while Nhan-Chang *et al*. (2010) compared ruptured and unruptured zones from the chorioamniotic membranes of the same individual. Liu *et al*. (2017) and Park *et al*. (2020) assessed ruptured and unruptured follicles using a genetic knockout model that exhibits an anovulatory phenotype. Additionally, Liu *et al*. (2017) used a zebrafish model, Park *et al*. (2020) a mouse model, while Kurki *et al*. (2011) and Nhan-Chang *et al*. (2010) presented data from humans. However, Liu *et al*. (2017) did note substantial overlap between zebrafish, human, and mouse pre-ovulatory data sets. Zebrafish are widely used in studies of reproductive function and fertility due to the relative ease of genetic manipulation and high genetic conservation with humans, estimated to be as high as 70% (Hajam *et al*., 2021; Santoriello & Zon, 2012). Although zebrafish do not exhibit the processes of COC expansion, luteinization, or implantation which occur in humans following ovulation, zebrafish ovaries contain follicles that rupture *via* core molecular signatures that are conserved in mammalian ovulation (Hajam *et al*., 2021). Park *et al*. (2020) used a mouse model and single-cell RNA sequencing that allowed specific analysis of the transcriptome of the mural granulosa cells that make up the rupturing follicle wall. However, this data set was limited to samples taken 6 h following induction of ovulation with hCG. In mice, complete follicle rupture occurs at around 12 h post-hCG stimulation, so this data set may be missing key transcriptomic signatures of late follicle rupture (Smith *et al*., 2022).

## IV. FEATURES OF INTRACRANIAL ANEURYSM FORMATION AND RUPTURE THAT PARALLEL OVULATION

We identified 120 genes that were upregulated or downregulated in both the Kurki *et al*. (2011) ICA and Liu *et al*. (2017) ovulation data sets (Fig. 2A), and 43 genes that were upregulated and downregulated in common for Kurki *et al*. (2011) and the Park *et al*. (2020) ovulation data set. These shared transcripts could provide potential targets for future studies in either system. ICAs and ovulation also share unique features other than their transcriptomic signatures. These rupture systems have similarities in immune cell infiltration and biomechanical fluid forces involved in formation of the rupture site and that contribute to eventual rupture.

### (1) Differentially expressed genes shared between ICAs and ovulation

Twelve genes were upregulated or downregulated in common among the two ovulation data sets and the ICA data set (Table 2; see Table S1 for the complete list of shared genes for each comparison). Of these 12 overlapping transcripts, 11 were upregulated in the ruptured group compared to the unruptured group. Two of these, *Angptl4* and *Pfkfb4*, were also found in the chorioamniotic membrane rupture data set (see Section III.1).

Of the remaining nine, spermidine/spermine N1-acetyltransferase 1 (*Sat1*) is upregulated in response to hCG in both bovine and human granulosa tissue (Lussier *et al*., 2017; Poulsen *et al*., 2020). It has also been characterized in thoracic aortic aneurysm rupture as part of the ferroptosis pathway and plays a role in the response to intracerebral haemorrhage following ICA rupture (Ren *et al*., 2022; Wang *et al*., 2022). Uncoupling protein 2 (*Ucp2*) is reduced in cases of impaired ovulation associated with fetal exposure to hypothyroidism and mediates apoptosis, gap junction integrity, and progesterone synthesis in cumulus cells (Ge *et al*., 2017; Meng *et al*., 2016; Rousset *et al*., 2003). In the context of ICAs, it is upregulated within the ruptured region of aneurysm walls and in cerebral ischemia following haemorrhage (Deierborg Olsson *et al*., 2008; Kurki *et al*., 2011). Regulator of G protein signalling 2 (*Rgs2*), NFKB inhibitor alpha (*Nfkbia*), and ADAM metallopeptidase domain 8 (*Adam8*) all increase in response to the hormonal trigger of ovulation in multiple mammalian models (Hernandez-Gonzalez *et al*., 2006; Hughes & Murphy, 2021; Kim, Bagchi & Bagchi, 2009; Paciolla *et al*., 2011; Park *et al*., 2020; Sayasith, Sirois & Lussier, 2014; Sriraman *et al*., 2008; Ujioka *et al*., 2000). *Nfkbia* and *Adam8* are poorly understood in the context of aneurysm rupture, but *Rgs2* is known to be upregulated in response to high wall shear stress and is associated with development of hypertension, which is a major risk factor for ICA development and rupture (Cañes *et al*., 2021; Dolan *et al*., 2012; Heximer *et al*., 2003; Leng *et al*., 2021; Tang *et al*., 2003). ATP binding cassette subfamily A member 1 (*Abca1*) is part of the peroxisome proliferator-activated receptor gamma (PPAR-gamma) pathway that mediates cholesterol influx in the ovary, is increased in early luteal stages, and is associated with altered fertility when mutated (Horihata *et al*., 2017; Miettinen, Rayburn & Krieger, 2001; Puttabyatappa, VandeVoort & Chaffin, 2010). It plays a similar cholesterol-transporting role in arterial walls (Bekelis *et al*., 2016; Ollikainen *et al*., 2016; Synowiec *et al*., 2016). Cholesterol processing plays a role in driving risk of rupture, and some variants of *Abca1* have been shown to be associated with decreased aneurysm risk (Bekelis *et al*., 2016; Ollikainen *et al*., 2016; Synowiec *et al*., 2016). Glutamate-ammonia ligase (*Glul*), bromodomain adjacent to zinc finger domain A1 (*Baz1a*), and DEAD-box helicase 3 X-linked (*Ddx3x*) have been characterized in some reproductive contexts, but there are no published studies establishing a role during ovulation (Bonnet *et al*., 2008; Pan *et al*., 2006; Yu *et al*., 2019). Conversely, there is evidence for involvement of three transcripts in the context of aneurysm formation, rupture, or response to rupture (Arnold *et al*., 2017; Krischek *et al*., 2008; Li *et al*., 2017; Yan *et al*., 2021; Zhong *et al*., 2022; Zhou *et al*., 2022). MAGUK P55 scaffold protein 7 (*Mpp7*), the only transcript downregulated in the ruptured group, is responsive to oestrogen receptor signalling in granulosa cells and responsive to versican in cumulus cells (Binder *et al*., 2013; Dunning *et al*., 2015). It was additionally found to be enriched in aneurysms generally compared to control vascular tissue (Pera *et al*., 2010; Zhong *et al*., 2021).

Although the list of overlapping genes for the ovulation–ICA comparisons is relatively small, the transcripts that are present in only one of these comparisons may also yield useful insights. One of the top enriched transcripts in ruptured tissue that is present in both Kurki *et al*. (2011) and Liu *et al*. (2017) is pentraxin 3 (*Ptx3*) (Table S1). This gene is reported to play an essential role in cumulus cell expansion, i.e. expansion of the layer of specialized granulosa cells around the oocyte that is required for ovulation and fertilization (Baranova *et al*., 2014; Richards *et al*., 2008; Salustri *et al*., 2004). *Ptx3*-knockout mice are subfertile but produce oocytes that are capable of *in vitro* fertilization, suggesting that its expression is essential for ovulation (Garlanda *et al*., 2002; Russell & Robker, 2007; Salustri *et al*., 2004; Varani *et al*., 2002). In ICAs, *Ptx3* has been most widely characterized in the context of subarachnoid haemorrhages, a potentially fatal consequence of ICA rupture. Elevated levels of *Ptx3* after subarachnoid haemorrhages are associated with vasospasm and increased mortality (Argın *et al*., 2017; Kati *et al*., 2020; Zanier *et al*., 2011). Although questions remain due to its reported presence in only one ovulation study in our comparisons, its presence in at least one comparison suggests that the overlapping gene lists may capture known regulators of the process of rupture in addition to novel transcripts of interest.

Because many of these overlapping transcripts participate in pathways driving ovulation and in aneurysm formation, rupture, and response to rupture, these transcripts warrant further experimental investigation. Future studies could investigate the roles of the transcripts listed in Table 2 in driving the process of follicle rupture. The additional 108 and 31 genes generated from the two ICA–ovulation comparisons (Fig. 2A) may yield additional targets for study with roles in the process of rupture during ovulation (Table S1).

### (2) Patterns of immune cell infiltration in ICAs and ovulation

Immune-related processes are known to be involved in both the ruptured vascular wall and ruptured follicles. Immune-related pathways may include the infiltration of external immune cells into the rupture system. Ruptured ICAs have larger populations of neutrophils, monocytes, M0 macrophages, and M2 macrophages (Shan *et al*., 2021). Most leukocytes that infiltrate aneurysmal walls are macrophages, and mice with depleted macrophages had a lower incidence of aneurysms (Kanematsu *et al*., 2011). Mast cells play a key role in aneurysm rupture and stabilization of mast cell degranulation may be a potential therapeutic option for preventing rupture (Furukawa *et al*., 2020). Similar classes of immune cells have been characterized in the ovary at the time of ovulation. Mast cells are of particular significance in the ovary as they produce three substantial mediators of ovulation: histamine, serotonin, and interleukin-8 (IL-8) (Morikawa *et al*., 1981; Szukiewicz *et al*., 2007). Within the ovary, serotonin stimulates progesterone production and plays a role in ovulation induction (Schmidt *et al*., 1988; Terranova *et al*., 1990). Histamine also plays a role in ovulation, possibly through stimulation of contractile cells within the follicle and through vasodilatory effects on follicle vasculature (Schmidt, Kannisto & Owman, 1990; Schmidt *et al*., 1987; Wallach, Wright & Hamada, 1978). The cytokine IL-8 stimulates an immune response, largely through recruitment of neutrophils (Szukiewicz *et al*., 2007). Mast cells also produce other enzymes such as tryptase and chymase that may contribute to ECM degradation leading up to ovulation (Brännström & Enskog, 2002).

### (3) Involvement of biomechanical forces in ICA and ovulation

The biomechanical forces and structural changes that contribute to ICA formation and rupture have been well characterized by studies investigating the pathophysiology of ICAs and stratification of patient risk. ICAs form in conditions of high neurovascular wall shear stress (WSS) paired with a weakened vascular endothelium due to cigarette smoke exposure, genetic disorders, or chronic hypertension (Keedy, 2006). WSS is a quantification of the force per unit area of fluid on a vascular wall and represents the mechanical forces to which the vascular endothelial cells are exposed (Papaioannou & Stefanadis, 2005). When high WSS is present in intracranial vessels, it causes endothelial cell damage and turnover, ECM degradation, medial thinning, and mural cell apoptosis (Dolan *et al*., 2012; Jamous *et al*., 2005; Kolega *et al*., 2011; Meng *et al*., 2011; Wang *et al*., 2009). These structural changes resulting from high WSS lead to changes in vessel geometry that facilitate aneurysm formation (Kulcsár *et al*., 2011; Shojima *et al*., 2004; Zhang *et al*., 2016). Once formed, the type and magnitude of WSS changes substantially in different regions of the aneurysm (Kawaguchi *et al*., 2012; Shimogonya *et al*., 2009). After initiation, aneurysms exhibit a decrease in the magnitude of WSS within the aneurysm compared to the adjacent non-aneurysmal vessel and a concomitant increase in oscillatory shear index (OSI), a non-dimensional parameter used to quantify disturbances in flow (Kawaguchi *et al*., 2012; Meng *et al*., 2014). By contrast, the magnitude of WSS at the point at which the aneurysm connects to the rest of the blood vessel is high (Kawaguchi *et al*., 2012; Meng *et al*., 2014). This indicates that there are distinct regions within the aneurysm: a region of lower WSS and high OSI that may rupture and produce a haemorrhagic stroke and a region of high WSS at the mouth of the aneurysm that is not prone to rupture.

Low WSS leads to structural changes and remodelling that predispose aneurysms to rupture. Low WSS paired with high OSI leads to a proinflammatory state with increased reactive oxygen species, immune cell infiltration, and matrix metalloproteinase (MMP) production by inflammatory cells (Galis *et al*., 1994; Gui *et al*., 2012; Malek, Alper & Izumo, 1999; Ross, 1999). Low WSS also leads to endothelial cell disruption and smooth muscle proliferation and migration (Chiu & Chien, 2011; Frosen *et al*., 2012). A certain magnitude of WSS is also necessary to maintain the level of remodelling needed for homeostatic maintenance of vessel walls (Chiu & Chien, 2011). When the WSS falls below this cut-off, the remodelling becomes deleterious and cellular elements of the vascular wall undergo apoptosis (Chiu & Chien, 2011).

Additionally, the magnitude of WSS and OSI within the aneurysm has an impact on rupture risk. Use of computational modelling with patient imaging data from 210 ICAs determined that maximum WSS was higher in ruptured aneurysms relative to unruptured aneurysms (Cebral *et al*., 2011). Another study similarly found that WSS averaged 2.92 N/m^2^ in ruptured aneurysms relative to 1.48 N/m^2^ in unruptured aneurysms, although both values were lower than the measured WSS in non-aneurysmal vessels (Shojima *et al*., 2004). This suggests that low WSS may lead to remodelling that weakens the wall of the aneurysm but that the WSS and OSI must still rise to a certain level to provide the necessary force for rupture.

Although the WSS associated with ovulation has not been well characterized, similar factors may contribute to follicle rupture. The follicle rupture site in hamster follicles undergoes three stages of structural and biomechanical changes during ovulation induction (Martin & Talbot, 1987). The first stage, occurring in the first 8 h following ovulation induction, involves substantial swelling of the antral cavity and uniform thinning around the entire follicle (Martin & Talbot, 1987). The second stage, over the next 4 h, is characterized by halted antral cavity growth and degeneration of the apical region of the follicle (Martin & Talbot, 1987). Finally, in the last 2 h, the basal region of the follicle thickens, and the apical region breaks apart and ruptures (Martin & Talbot, 1987). Early studies of intrafollicular pressure did not identify a substantial change within ovulatory follicles, but they noted that the follicle wall changes in thickness and extensibility which may alter the wall stress and contribute to additional remodelling that leads to rupture (Espey & Lipner, 1963; Rondell, 1964). A more recent study found a substantial increase in intrafollicular pressure after ovulation induction (Matousek *et al*., 2001). Additionally, the driving force of intrafollicular pressure is directly related to blood flow within the follicle (Rodgers & Irving-Rodgers, 2010). Use of transvaginal ultrasonography with colour Doppler imaging to visualize regional blood flow in human follicles identified increased blood flow to the follicle base and decreased blood flow to the follicle apex during ovulation (Brannstrom *et al*., 1998). Although the intrafollicular pressure is generally increased in the follicle after ovulation induction, this pressure may be asymmetrically distributed and thus may have regions of differing WSS that could play a role in formation of the rupture zone.

Biomechanical changes are also similar between follicle rupture and rupture of ICAs. Both systems have an early phase of high pressure and WSS followed by a period of lower biomechanical forces that promote remodelling and predispose them to rupture. Novel methods for studying ovulation and follicle rupture in a controlled manner, such as *in vitro* follicle growth and rupture assays, will be important to understand the biomechanics of follicle rupture at a higher resolution (Skory *et al*., 2015). Understanding patterns of altered blood pressure may have implications for luteinized unruptured follicle syndrome, a condition that is often incidentally identified in cases of unexplained infertility associated with follicles that fail to rupture despite being able to form a corpus luteum (Marik & Hulka, 1978). A case report of medication-induced luteinized unruptured follicle syndrome in patients taking paracetamol, an over-the-counter pain medication, found that there was reduced systolic blood velocity in these unruptured follicles as a result of vasodilation (Bourne *et al*., 1991). The mechanism of luteinized unruptured follicle syndrome has yet to be comprehensively characterized (Marik & Hulka, 1978). WSS modelling and fluid flow analysis techniques derived from studies of ICAs could provide novel insights into the basic biology of ovulation and ovulation-associated pathologies.

The study of ICAs has also generated novel methods that could be used to understand ovarian follicle rupture better. For example, four-dimensional (4D) flow-imaging modalities have been used to assess fluid dynamics during aneurysm formation, growth, and rupture (Brindise *et al*., 2019; Medero *et al*., 2020; Vivas *et al*., 2022). The data from this imaging are used to create *in silico* models of how changes in flow affect progression of the aneurysm. Microfluidic models have also been adapted to assess how fluid shear stress alters the morphology and transcriptomic signatures of vascular endothelial cells *in vitro* (Doherty *et al*., 2021). Ovulation studies could benefit from high-resolution modelling of the impact of fluid pressures and flows on formation of a rupture site. Recently, optical coherence tomography (OCT), which is used to assess aneurysm structure and model flow dynamics, has been adapted to reproductive biology contexts (Hartmann *et al*., 2019; Liu *et al*., 2019; Real *et al*., 2013; Umezu & Larina, 2022). Intravital OCT was used to study COC transit through the oviduct following ovulation and more recently to visualize follicle rupture and transport of COCs through the ovarian bursa to the oviduct (Burton *et al*., 2015; Umezu, Wang & Larina, 2022). Additional modalities, including doppler-equipped intravital imaging, could be used to assess specific fluid dynamics within the ovulatory follicle. These data could then be used to assess the role that fluid-derived biomechanical forces may play in follicular wall rupture. Additionally, microfluidic studies using flow metrics generated from the imaging data could assess the impact of fluid shear forces on the structure and function of granulosa cells derived from follicle walls. These studies could use strategies from ICA modelling, such as generating three-dimensional (3D)-printed scaffolds that recapitulate ovarian follicle geometry (Doherty *et al*., 2021; Jamous *et al*., 2005; Vivas *et al*., 2022). Microfluidic technology could also be used in ovulation to assess how fluid dynamics change the structure and function of granulosa cells.

## V. FEATURES OF CHORIOAMNIOTIC MEMBRANE RUPTURE THAT PARALLEL OVULATION

The comparison between the data sets of Nhan-Chang *et al*. (2010) and Liu *et al*. (2017) revealed 48 genes that were upregulated or downregulated in common (Fig. 2B), while the comparison between Nhan-Chang *et al*. (2010) and Park *et al*. (2020) revealed 18 genes that were upregulated or downregulated in common. Some of these transcripts have already been characterized in chorioamniotic membranes and ovulation. In addition to the transcriptomic data from chorioamniotic membrane rupture, other features both physiologically at term and in cases of pathological preterm labour parallel ovulation, including altered smooth muscle cell contractility and immune cell infiltration.

### (1) Differentially expressed genes shared between chorioamniotic membrane rupture and ovulation

The three upregulated genes found in both ovulation data sets and in the chorioamniotic membrane rupture data set are listed in Table 3 (see Table S2 for the complete list of shared genes for each comparison). Two of the three upregulated genes, *Angptl4* and *Pfkfb4*, were also identified in the ovulation–ICA comparisons (see Section IV.1). Since ovulation and chorioamniotic rupture both represent physiological forms of rupture, it is of interest to consider the other identified overlapping gene, *Lox*, which was not present in the ovulation–ICA comparisons and thus may be involved in processes that are unique to physiological rupture. Lysyl oxidase (Lox) functions in the crosslinking of collagens and elastin (Lucero & Kagan, 2006). *Lox* is responsive to several regulators of ovulation, including transforming growth factor beta 1 (TGF-ß1), follicle stimulating hormone (FSH), growth differentiation factor 9 (GDF-9), and Activin A in both human and rat granulosa cells (Fang *et al*., 2016; Harlow *et al*., 2003; Slee *et al*., 2001). *Lox* has also been shown to play a role in remodelling associated with rupture of chorioamniotic membranes during parturition (Polettini *et al*., 2016). Specifically, it is responsive to cortisol produced by the amnion (Liu *et al*., 2016*a*). It also participates in cyclooxygenase-2 pathways and prostaglandin E2 signalling in fetal membranes to regulate the breakdown of membranes during rupture (Liu *et al*., 2016*b*). Although relatively few transcripts overlapped completely between the two ovulation and chorioamniotic rupture comparisons, there were 60 transcripts that overlapped in only one of these two comparisons (Fig. 2B, Table S2). One of these transcripts is prostaglandin-endoperoxide synthase 2 (*Ptgs2*), which was upregulated in the ruptured tissue in both Nhan-Chang *et al*. (2010) and Liu *et al*. (2017). *Ptgs2* is a major driver of ovulation that is temporally expressed during the LH surge and downregulated shortly afterwards (Park *et al*., 2020; Richards, 1997; Wong & Richards, 1991). Functional blocking of Ptgs2 impairs ovulation and has been proposed as a potential contraceptive (Armstrong & Grinwich, 1972; Downs & Longo, 1982; Duffy, 2015; Mikuni *et al*., 1998; Sogn *et al*., 1987). In chorioamniotic membranes, *Ptgs2* regulates the activity of MMPs that facilitate membrane rupture (Li *et al*., 2007). *Ptgs2* messenger RNA (mRNA) is upregulated in term-labour relative to non-labour membranes (Takahashi *et al*., 2021). Although this transcript was not identified in the comparison between Nhan-Chang *et al*. (2010) and the other ovulation data set (Park *et al*., 2020) and is thus not a robust finding, its presence in at least one of our comparisons implies that it may be involved in the process of rupture. Further research into the genes identified in Table 3 and Table S2 may elucidate novel regulators of follicle rupture during ovulation, and more generally in processes that may be unique to physiological rupture.

### (2) Patterns of immune cell infiltration in chorioamniotic membrane rupture and ovulation

Immune cell infiltration plays a major role in chorioamniotic membrane rupture. Intrauterine infection increases the likelihood of preterm chorioamniotic membrane rupture and is associated with immune cell infiltration. Neutrophils are associated with preterm labour in the setting of intrauterine infection (Hamilton *et al*., 2012). Macrophages are also present in different regions of the uterus and chorioamniotic membranes leading up to parturition (Bokström *et al*., 1997). Macrophages facilitate remodelling of the supracervical region of the chorioamniotic membranes that leads to weakening and predisposes this region to rupture during parturition (Hamilton *et al*., 2012; Mackler *et al*., 1999). This weakening is controlled largely by activation of macrophage-derived MMPs that are major drivers of ECM remodelling and degradation (Gonzalez *et al*., 2011; Payne *et al*., 2012). Macrophages also play a role in remodelling after membrane rupture (Shynlova *et al*., 2013; Timmons, Akins & Mahendroo, 2010). T lymphocytes are another key immune mediator in chorioamniotic membrane rupture. In term parturition, these cells infiltrate the chorioamniotic membranes and are concentrated at the rupture zone (Gomez-Lopez *et al*., 2011, 2013*b*). However, in preterm rupture of membranes, there is less infiltration by T lymphocytes and more by granulocytes (Gomez-Lopez *et al*., 2013*a*,b). This suggests that alterations in immune cell infiltration may play a role in pathological forms of chorioamniotic membrane rupture.

These patterns of immune cell infiltration parallel features of ovulation where macrophage infiltration occurs in the vasculature and theca cell layer (Brännström & Enskog, 2002; Brannstrom, Mayrhofer & Robertson, 1993). Macrophage density increases in the corpus luteum after ovulation, suggesting they may play a similar role in repairing tissue after rupture in the ovulatory follicle (Brannstrom *et al*., 1994; Cavender & Murdoch, 1988). T lymphocyte infiltration also occurs in ovulatory follicles, particularly in the theca vasculature (Best *et al*., 1996). Interestingly, the number of T lymphocytes is significantly decreased in infertile women with polycystic ovarian syndrome (Li *et al*., 2019). In both chorioamniotic membrane rupture and ovulation, pathological forms of rupture are associated with alterations in T lymphocyte counts.

### (3) Involvement of smooth muscle contraction in chorioamniotic membrane rupture and ovulation

Biomechanical forces play a role in the rupture of chorioamniotic membranes, both at term and in pathological preterm rupture, although the exact mechanism may be variable. Early research hypothesized that uterine contractions during labour lead to chorioamniotic membrane rupture through mechanical stretching and tearing of the membranes (Joyce *et al*., 2009). However, this cannot be the only mechanism as rupture occurs prior to labour-associated contractions in 10% of term deliveries and 40% of preterm deliveries (Joyce *et al*., 2009). Additionally, an *in vitro* study of chorioamniotic membrane strength found that acute mechanical stretching in fact strengthens chorioamniotic membranes (Pandey *et al*., 2007). It is now thought that biomechanical stretch plays a role in chorioamniotic membrane rupture through an alternative mechanism: induction of inflammation and remodelling.

Chorioamniotic membranes have a well-characterized weak zone above the cervical region that forms prior to rupture (Kumar *et al*., 2016). The weakened zone forms due to disruption of collagen, increased collagenase activity, and inflammation (Kumar *et al*., 2016). This remodelling occurs prior to labour, the point in pregnancy at which it is subjected to the highest mechanical stretching force (Kumar *et al*., 2016). The weakened zone is found even in chorioamniotic membranes from caesarian sections that have not undergone labour (McLaren *et al*., 1999). There is evidence that it is not the acute, high-magnitude forces produced by contractions that lead to this weakened zone but rather it is the smaller, cyclical forces from smooth muscle cell contractions that occur throughout gestation (Kumar *et al*., 2016; Pandey *et al*., 2007). Chronic stretching of chorioamniotic membranes, particularly in the region overlying the cervix, induces IL-8 signalling that dictates remodelling of the region (Maehara *et al*., 1996). Increased *Cox2* expression, prostaglandin E2 (PGE2) release, and collagenase activity occurs following mechanical stretching of chorioamniotic membranes (Chowdhury *et al*., 2014; El Maradny *et al*., 1996). Chronic stretching of the chorioamniotic membranes as part of gestation is also associated with inflammation, ECM remodelling, and some degree of protection from apoptosis until shortly before delivery (Kendal-Wright, Hubbard & Bryant-Greenwood, 2008). Mechanical deformation of collagen in the chorioamniotic membrane makes the collagen more susceptible to degradation by stretching the fibres and exposing more crosslinking sites to collagen-degrading enzymes (Joyce *et al*., 2009). Once the weakened zone is formed, rupture may be triggered either by mechanical forces of labour or by an increased inflammatory state, often implicated in pathological ruptures of chorioamniotic membranes.

Smooth muscle contractions also play a role in ovulation, with a series of morphological changes occurring in the ovulatory follicle (Martin & Talbot, 1981*b*). Smooth muscle cells are only present in the basal region of the follicle, particularly in the theca externa layer, and this region of the follicle is constricted at the time of rupture (Martin & Talbot, 1981*b*). In hamster follicles, a substantial decrease in intrafollicular pressure coincides with contraction of the smooth muscle cells at the follicle base (Schroeder & Talbot, 1982). Within human periovulatory follicles, non-vascular smooth muscle cells are found in the theca externa and expression of key components of the endothelin system which regulates contractions is high (Choi *et al*., 2011).

There is evidence for smooth muscle involvement in ovulation. In one study of *in vivo* ovulation in a hamster model, drugs that inhibit smooth muscle contraction blocked ovulation (Martin & Talbot, 1981*a*). Drugs that alter calcium availability within the follicle also inhibited ovulation due to the involvement of calcium transport in smooth muscle contraction (Martin & Talbot, 1981*a*). Endothelin-2 induces smooth muscle contraction, and an endothelin2-knockout mouse model exhibited reduced rates of ovulation and luteinization (Cacioppo *et al*., 2017). However, there are no studies that link biomechanical force production by smooth muscle contraction in the theca externa to changes in structure and pathway expression of the apical region of the follicle. In *ex vivo* models of rupture of chorioamniotic membranes, manipulation of mechanical stretch forces exerted on chorioamniotic membranes had a direct impact on pathways related to inflammation and ECM remodelling (Chowdhury *et al*., 2014; El Maradny *et al*., 1996; Joyce *et al*., 2009; Kendal-Wright *et al*., 2008; Maehara *et al*., 1996). One study of human cervical fibroblasts cultured on plates with flexible silicon bottoms found that cyclical mechanical stretch forces led to increased production of hyaluronan, which is necessary for cervical ripening during parturition (Takemura *et al*., 2005). Smooth muscle-like contraction as well as hyaluronan production, largely by cumulus cells within the ovulatory follicle, are both essential for ovulation (Choi *et al*., 2011; Hess, Chen & Larsen, 1999; Martin & Talbot, 1981*a*,b; Richards, 2005; Russell & Robker, 2007; Talbot & Chacon, 1982). Adapting *ex vivo* stretching studies from the study of parturition may provide evidence for an additional mechanism that drives alterations in ECM composition and molecular signatures of ovulation within the ovulatory follicle. Further analysis of the role of smooth muscle contraction and mechanical stretch within periovulatory follicles using *ex vivo* application of mechanical force and stretch forces to cells isolated from the follicle wall may provide additional insights into fundamental mechanisms of ovulation as well as elucidate potential targets for contraceptive development.

Due to the significant role of mechanical stretch in chorioamniotic membrane rupture, several methods have been developed to introduce stretch forces to membranes *in vitro* and assess how morphology, ECM composition, and transcriptomic signatures change with stretching (Chowdhury *et al*., 2014; Kumar *et al*., 2016). In addition, a puncture-testing method has been adapted from the textile industry to assess how small forces and stretch distend the chorioamniotic membrane to study the biomechanical properties of this tissue (Burzle, Mazza & Moore, 2014). Testing of mechanical stretch in the context of ovulation and the ovary could also elucidate mechanisms of impaired ovulation associated with advanced reproductive age. There is evidence for defects in follicle rupture associated with ovarian aging, demonstrated by an increased incidence of antral follicles with expanded cumulus oocyte complexes that have failed to rupture along with oocytes trapped in luteinized unruptured follicles (Mara *et al*., 2020). There is also evidence for impaired epithelial layer remodelling and wound healing as well as altered proliferation and apoptosis (Mara *et al*., 2020). These studies were performed in mice treated with pregnant mare serum gonadotropin (PMSG), a FSH analogue, prior to induction of ovulation with hCG, a LH analogue. This hyperstimulation and superovulation led to recruitment and ovulation of a larger number of follicles in one cycle. The finding that ovulatory defects are observed with advanced age even in the context of superovulation demonstrates that these defects are driven by factors other than age-related changes in the hormonal stimulation of ovulation. The mechanisms for these phenotypes are still under investigation but could be due to increased inflammation and stromal fibrosis, and subsequent ovarian tissue stiffness, which is known to increase with age (Amargant *et al*., 2020; Mara *et al*., 2020). Acute treatment with anti-fibrotic agents improves ovulation outcomes with advanced reproductive age (Umehara *et al*., 2022). Previous studies in the ovary have used instrumental indentation, atomic force microscopy, and shear wave elastography techniques to quantify stiffness (Amargant *et al*., 2020; Ansardamavandi *et al*., 2020; Gargus *et al*., 2020; Sumbul *et al*., 2022). Additional techniques used to study cervical and chorioamniotic membrane changes during parturition could also be adapted to the study of ovulation. These include Raman spectroscopy, which has been used non-invasively in *in vivo* studies of cervical remodelling and avoids the necessity for invasive measurements of biomechanical properties of the cervix (O’Brien *et al*., 2017). Other techniques may include studies of tissue deformability using aspiration distension or strain (static) elastography to assess the distensibility of the ovarian follicle wall, both of which have been used in studies of parturition (Chowdhury *et al*., 2014; Feltovich & Carlson, 2017). Understanding how aging impacts distensibility of ovarian follicle walls may provide insights into the mechanisms behind age-associated follicle rupture defects.

Studies of chorioamniotic membrane rupture have increased our understanding of transcriptomic signatures around the time of rupture. Many studies have identified differences in signalling pathways between the supracervical rupture site compared to sites distant to rupture (Arikat *et al*., 2006; Lappas *et al*., 2008; Moore *et al*., 2009). This technique could be adapted to the study of follicle rupture by using spatial transcriptomics in ovulatory follicles across the time course of ovulation. It could also be used to isolate transcriptomic signatures of tissue specific to the region of follicle rupture as well as tissue from a region distant to the rupture site in ovulatory follicles. Together, methods for testing the role of mechanical stretch forces on granulosa cells as well as spatial transcriptomic studies of rupture in ovulation will enable us to understand the process of rupture at high resolution.

## VI. DISCUSSION

**Fig. 3.**
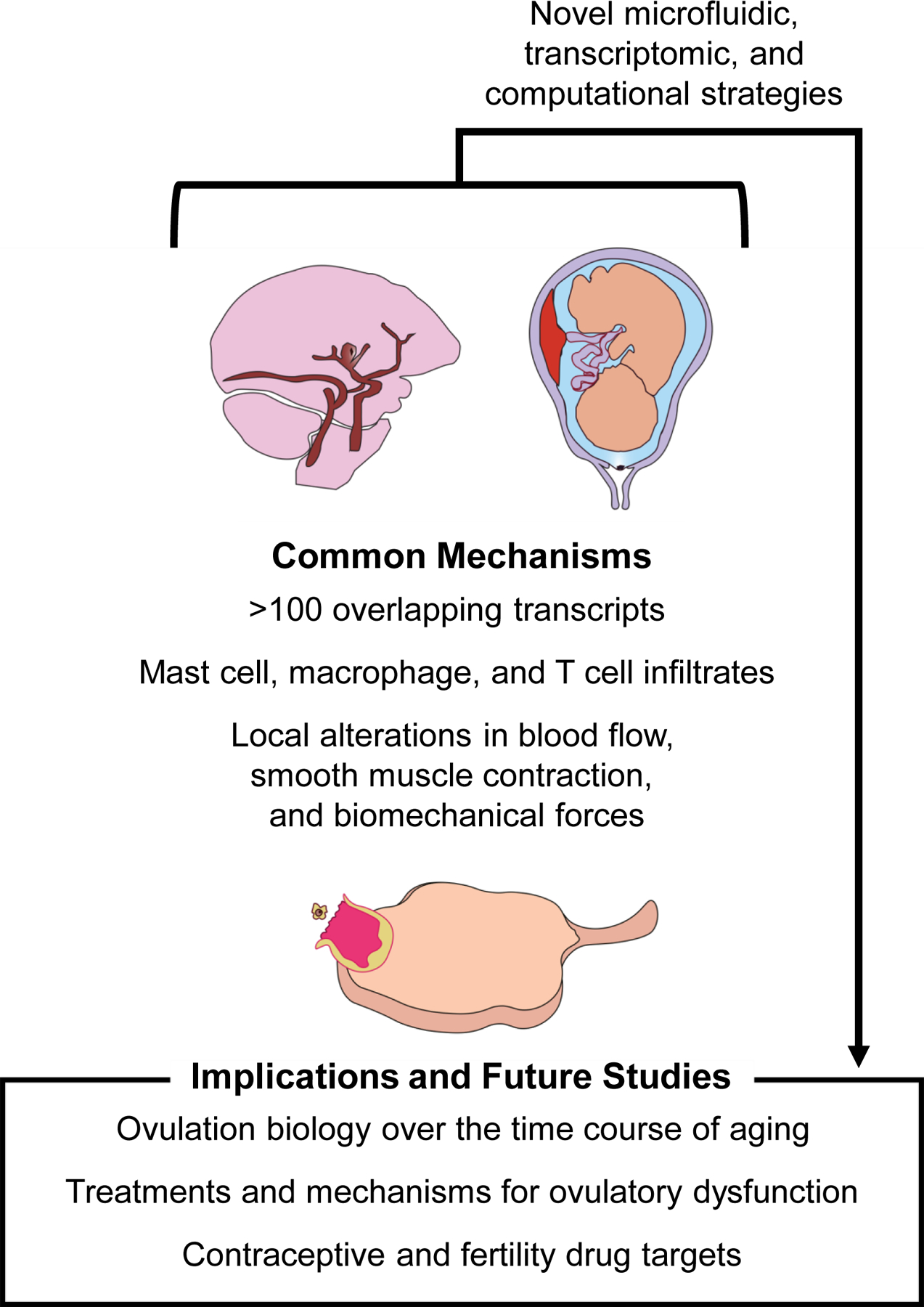
Understanding common mechanisms of rupture between pathological and physiological rupture systems in addition to novel methods of studying rupture can be used to inform potential innovations in studies of ovarian biology and ovulation.

The observed parallels in the transcriptomic signatures, immune profiles, and structural forces that underlie rupture in ICAs, chorioamniotic membranes during parturition, and ovarian follicles during ovulation extend our understanding of ovulation (Fig. 3). The current understanding is that ovulation involves ECM remodelling, inflammation, and structural forces that drive follicle rupture. Comparing biology across pathological and physiological examples of rupture in the human body demonstrates that these general pathways are conserved. In addition, these comparisons highlight key genes that are conserved across multiple comparisons, including *Angptl4*, *Pfkfb4*, and *Lox*. Several of the identified genes have been characterized previously in rupture contexts, whereas others, including *Glul*, *Baz1a*, and *Ddx3x*, are not yet characterized and should be explored further in the context of ovarian follicle rupture. Comparisons of the biomechanical and structural forces that underlie rupture also identify broad similarities in WSS decreases leading up to rupture and mechanical stretch of smooth muscle-like cells away from the site of rupture. The role that these forces play and their potential for clinical and diagnostic use is better understood in the context of ICA and chorioamniotic membrane rupture than in ovulation. As a result of the similarities between rupture systems discussed herein, *ex vivo* methods that have been developed to study ICAs and chorioamniotic membrane rupture should be paired with existing *ex vivo* ovulation techniques to advance the field of ovulation biology (Converse *et al*., 2022; Skory *et al*., 2015). The gaps identified in this review, specifically in the context of the biomechanical and contractile forces involved, should be explored further to enhance our understanding of the process of ovulation.

Novel insights may arise from a deeper investigation of poorly characterized genes from this transcriptomic data set, the role of immune cells and immune modulation, and the impact of biomechanical forces on the process of follicle rupture, particularly with reference to the pathology of conditions associated with abnormal or impaired follicle rupture such as luteinized unruptured follicle syndrome and ovarian aging (Mara *et al*., 2020; Marik & Hulka, 1978). Learning more about ovulation may elucidate drug targets for ovarian pathologies as well as the development of ovulation-modifying drugs, such as non-hormonal contraceptives. Although the process of ovulation is exceedingly complex, many of its features are reflected in other processes that can be described as rupture events.

Our analysis suggests that ICAs and chorioamniotic membrane rupture share several transcriptomic and non-transcriptomic features with ovulation. Given these parallels between rupture systems and ovulation, there should be a focus on sharing insights to advance each of these fields. Much work has been done to understand rupture in the context of ICAs and chorioamniotic membranes in part because of the risk of morbidity and mortality in these conditions. Ovulation biologists should continue to examine findings from other rupture systems to identify relevant research questions and methods pioneered in other fields that study pathological and physiological rupture.

While such comparisons are compelling, they are not without caveats. First, comparisons of DEGs may not be as effective as direct transcriptomic comparisons of sequencing data. Future studies of the genes identified in this analysis should include validation of findings from the referenced transcriptomic data sets. Second, the designs of the transcriptomic analyses used herein differed in some respects (e.g. comparing tissues from the same individual/organism *versus* two different organisms; generating ruptured/unruptured phenotypes using knockout models *versus* using patient samples; or using RNA-Seq *versus* microarray techniques). Third, there are fundamental differences in the biology of these systems, specifically in relation to timing and pathophysiology. In humans, ovulation occurs over a period of hours, and while rupture of fetal membranes also takes place on this timescale, the formation of a rupture site may begin earlier in gestation. By contrast, rupture of ICAs occurs over a matter of years, or may not happen at all. Despite these differences in timing, the rupture events follow a similar pattern: initiation, growth and development of the rupture site, and finally rupture. With regard to pathophysiology, ovulation differs from the other rupture systems in that it is a purely physiological process. ICAs are always pathological whereas chorioamniotic membrane rupture can be pathological or physiological. Thus, while all rupture processes may not be the same, they do have some parallels that could be explored to further our understanding of each rupture system.

## VII. CONCLUSIONS

1. Intracranial aneurysm (ICA) rupture, chorioamniotic membrane rupture, and ovulation can all be described as rupture events.
2. These rupture events have overlapping transcriptomic signals (*Angptl4*, *Pfkfb4*) relating to vascularization, lipid metabolism, apoptotic regulation, and hypoxia response.
3. Our transcriptomic comparison has identified several novel transcripts that have not yet been characterized in the context of ovulation, including *Glul*, *Baz1a*, and *Ddx3x*.
4. ICA and chorioamniotic rupture share overlapping structural and biomechanical features with ovulation, including spatially distributed wall shear stress in both ICAs and ovulation and smooth muscle contractions away from the rupture site in both chorioamniotic membrane rupture and ovulation.
5. While there are limitations to this analysis, overlapping findings between these rupture systems and ovulation suggest that knowledge from these fields may be used to inform novel studies on ovulation, ovarian disorders, and ovulation-related drug development.
6. Specific methods that have been developed in various rupture systems including microfluidic studies, computational flow modelling, biomechanical stretch testing, and spatially oriented transcriptomic comparisons may be useful to advance the study of ovulation.

## Supplementary Material

Table S1 Complete list of overlapping upregulated and downregulated genes in comparisons between two ovulation data sets and one data set for intracranial aneurysm (ICA).

Table S3 Complete list of overlapping upregulated and downregulated genes in comparisons between the intracranial aneurysm (ICA) and chorioamniotic membrane rupture (CMR) data sets.

Table S2 Complete list of overlapping upregulated and downregulated genes in comparisons between two ovulation data sets and one data set for chorioamniotic membrane rupture (CMR).

## Figures and Tables

**Table 1.** Overview of transcriptomic data sets selected for our comparison of three rupture systems.

Data set	Rupture System	Results	Method	Organism	Sample details	GEO accession number
Kurki *et al*. (2011)	Intracranial aneurysm (ICA)	1,426 DEGs between ruptured and unruptured ICA walls	Microarray	Human	11 ruptured and 8 unruptured saccular ICA vascular walls	GSE13353
Nhan-Chang *et al*. (2010)	Chorioamniotic membrane rupture	677 DEGs between the ruptured zone above the cervix and a distal site after spontaneous rupture of membranes at term	Microarray	Human	20 matched sets of tissue samples of the chorion and amnion from the site of chorioamniotic membrane rupture and a region distant from the rupture site	–
Liu *et al*. (2017)	Ovulation	2,888 DEGs between preovulatory follicles and anovulatory follicles from a *Pgr-*KO model	RNAseq	Zebrafish	Isolated follicular cells (theca and granulosa) from three wildtype and three *Pgr*-KO female zebrafish prior to ovulation	–
Park *et al*. (2020)	Ovulation	720 DEGs between mural granulosa cells from Esr2-*Pgr*-KO mice and wildtype mice 6 h after ovulation	Single-cell RNAseq	Mouse	Single cell dissociation of eight ovaries from wildtype C57BL/6 mice and *Esr2-Pgr-*KO mice 6 h after induction of ovulation with hCG, differentially expressed gene list based on ovulatory mural granulosa cells	GSE145107

GEO, gene expression omnibus; DEG, differentially expressed gene; Esr, estrogen receptor; hCG, human chorionic gonadotropin; KO, knockout; Pgr, progestin receptor; RNAseq, RNA sequencing.

**Table 2.** List of upregulated (+) and downregulated (−) genes in common among the two ovulation data sets and the intracranial aneurysms (ICA) data set.

Gene	Gene function	Ovulation (Liu *et al*., 2017) Log2FC	Ovulation (Liu *et al*., 2017) P-value	Ovulation (Park *et al*., 2020) Log2FC	Ovulation (Park *et al*., 2020) P-value	ICA (Kurki *et al*., 2011) Log2FC	ICA (Kurki *et al*., 2011) P-value
*Sat1*	N1-acetyltransferase	1.093	0.01	0.330	<0.0001	2.539	0.0002
*Glul*	Ligase	4.073	<0.0001	0.989	<0.0001	2.296	0.0003
*Ucp2*	Mitochondrial protein	3.170	<0.0001	0.940	<0.0001	2.195	0.01
*Rgs2*	GPCR regulator	7.205	<0.0001	0.821	<0.0001	1.922	0.01
*Angptl4*	Secreted protein	1.792	<0.0001	0.317	<0.0001	1.811	0.002
*Abca1*	ATP binding	3.389	<0.0001	0.312	<0.0001	1.736	0.007
*Baz1a*	Chromatin assembly	1.390	0.002	0.656	<0.0001	1.269	0.008
*Ddx3x*	Helicase	1.603	<0.0001	0.334	<0.0001	0.824	0.0007
*Nfkbia*	NFKB inhibitor	2.861	<0.0001	0.450	<0.0001	0.799	0.04
*Pfkfb4*	Kinase	2.596	<0.0001	0.447	<0.0001	0.705	0.04
*Adam8*	Metalloproteinase	3.550	<0.0001	0.811	0.03	0.623	0.05
*Mpp7*	Scaffold protein	−1.210	0.006	−0.325	<0.0001	−1.184	0.001

FC, fold change; GPCR, G-protein coupled receptor; NFKB, nuclear factor kappa beta.

Liu *et al*. (2017) and Kurki *et al*. (2011) denoted genes as upregulated in ruptured tissue relative to unruptured tissue. Park *et al*. (2020) used the opposite convention, with genes denoted as upregulated in unruptured relative to ruptured tissue. We therefore have transformed the results of Park *et al*. (2020) to reflect the convention used in the other studies for ease of comparison.

**Table 3.** List of upregulated and downregulated genes in common among two ovulation data sets and the chorioamniotic membrane rupture (CMR) data set.

Gene	Gene function	Ovulation (Liu *et al*., 2017) Log2FC	Ovulation (Liu *et al*., 2017) P-value	Ovulation (Park *et al*., 2020) Log2FC	Ovulation (Park *et al*., 2020) P-value	CMR (Nhan-Chang *et al*., 2010) Log2FC	CMR (Nhan-Chang *et al*., 2010) P-Value
*Angptl4*	Secreted protein	1.792	<0.0001	0.317	<0.0001	2.40	0.0009
*Lox*	Oxidase	1.924	0.0002	0.275	0.005	2.30	0.001
*Pfkfb4*	Kinase	2.596	<0.0001	0.447	<0.0001	1.50	0.009

FC, fold change.

Liu *et al*. (2017) and Nhan-Chang *et al*. (2010) denoted genes as upregulated in ruptured tissue relative to unruptured tissue. Park *et al*. (2020) used the opposite convention, with genes denoted as upregulated in unruptured relative to ruptured tissue. We therefore have transformed the results of Park *et al*. (2020) to reflect the convention used in the other studies for ease of comparison.
