# Supplementary material for: Common mechanisms of physiological and pathological rupture events in biology: novel insights into mammalian ovulation and beyond
Source: Biol Rev Camb Philos Soc. Author manuscript; Available in PMC 2023 Oct 1. (PMC10524764; doi:10.1111/brv.12970)
Supplement: Table S1 — Complete list of overlapping upregulated and downregulated genes in comparisons between two ovulation data sets and one data set for intracranial aneurysm (ICA). [file NIHMS1902325-supplement-Table_S1.docx]

**Table S1.** Complete list of overlapping upregulated and downregulated genes in comparisons between two ovulation data sets and one data set for intracranial aneurysm (ICA).

| **Gene** | **Ovulation (Liu *et al*., 2017) Log2FC** | **Ovulation (Liu *et al*., 2017)**  **P-value** | **Ovulation (Park *et al*., 2020)**  **Log2FC** | **Ovulation (Park *et al*., 2020)**  **P-value** | **ICA (Kurki *et al*., 2011) Log2FC** | **ICA (Kurki *et al*., 2011)**  **P-value** |
| --- | --- | --- | --- | --- | --- | --- |
| *Rgs2* | 7.21 | <0.0001 | 0.82 | <0.0001 | 1.92 | 0.01 |
| *Sgk1* | 6.33 | <0.0001 | – | – | 3.45 | 0.0006 |
| *Ptx3* | 4.68 | <0.0001 | – | – | 3.53 | 0.009 |
| *Psme4* | 4.66 | <0.0001 | – | – | 1.36 | 0.0004 |
| *Glul* | 4.07 | <0.0001 | 0.99 | <0.0001 | 2.30 | 0.0003 |
| *Mafb* | 3.99 | <0.0001 | – | – | 2.62 | 0.0005 |
| *Hif1a* | 3.69 | <0.0001 | – | – | 1.49 | <0.0001 |
| *Adam8* | 3.55 | <0.0001 | 0.81 | 0.03 | 0.62 | 0.05 |
| *Abca1* | 3.39 | <0.0001 | 0.31 | <0.0001 | 1.74 | 0.007 |
| *Rbm47* | 3.26 | <0.0001 | – | – | 1.07 | 0.006 |
| *Ucp2* | 3.17 | <0.0001 | 0.94 | <0.0001 | 2.20 | 0.01 |
| *Phc2* | 3.15 | <0.0001 | – | – | 0.74 | 0.03 |
| *Hsd3b7* | 3.15 | <0.0001 | – | – | 0.81 | 0.009 |
| *Grb10* | 3.12 | <0.0001 | – | – | 0.81 | 0.03 |
| *Impdh1* | 2.94 | <0.0001 | – | – | 1.45 | 0.002 |
| *Csf1r* | 2.92 | <0.0001 | – | – | 1.31 | 0.02 |
| *Nfkbia* | 2.86 | <0.0001 | 0.45 | <0.0001 | 0.80 | 0.04 |
| *Ubash3b* | 2.80 | <0.0001 | – | – | 0.79 | 0.003 |
| *Stk10* | 2.80 | <0.0001 | – | – | 1.32 | 0.003 |
| *Lrrc8d* | 2.79 | <0.0001 | – | – | 1.46 | 0.003 |
| *Hmox1* | 2.79 | <0.0001 | – | – | 3.33 | 0.0003 |
| *Slc7a5* | 2.69 | <0.0001 | – | – | 1.21 | 0.05 |
| *Tnfrsf21* | 2.66 | <0.0001 | – | – | 1.12 | 0.006 |
| *Pfkfb4* | 2.60 | <0.0001 | 0.45 | <0.0001 | 0.70 | 0.04 |
| *Trib1* | 2.51 | <0.0001 | – | – | 0.87 | 0.01 |
| *Mxd1* | 2.43 | <0.0001 | – | – | 1.03 | 0.02 |
| *Pla2g7* | 2.40 | <0.0001 | – | – | 1.10 | 0.02 |
| *Dab2* | 2.40 | <0.0001 | – | – | 1.06 | 0.004 |
| *Stk40* | 2.36 | <0.0001 | – | – | 0.53 | 0.03 |
| *Slc20a1* | 2.35 | <0.0001 | – | – | 1.29 | 0.01 |
| *Snx11* | 2.32 | <0.0001 | – | – | 0.57 | 0.03 |
| *Mcl1* | 2.32 | <0.0001 | – | – | 0.54 | 0.02 |
| *Crem* | 2.28 | <0.0001 | – | – | 0.63 | 0.03 |
| *Ptp4a2* | 2.13 | <0.0001 | – | – | 1.08 | 0.001 |
| *Gltscr1* | 2.13 | <0.0001 | – | – | 0.56 | 0.04 |
| *Mpp1* | 2.02 | <0.0001 | – | – | 1.74 | 0.0006 |
| *Neto2* | 2.01 | 0.0007 | – | – | 0.77 | 0.05 |
| *Actr2* | 1.97 | 0.001 | – | – | 0.68 | 0.02 |
| *Mmd* | 1.93 | <0.0001 | – | – | 1.25 | 0.03 |
| *Ccnl1* | 1.90 | <0.0001 | – | – | 0.82 | 0.05 |
| *Fes* | 1.88 | <0.0001 | – | – | 0.85 | 0.02 |
| *Tgif1* | 1.88 | <0.0001 | – | – | 1.16 | 0.0004 |
| *Dysf* | 1.85 | <0.0001 | – | – | 0.84 | 0.05 |
| *Fam49a* | 1.79 | <0.0001 | – | – | 1.05 | 0.04 |
| *Angptl4* | 1.79 | <0.0001 | 0.32 | <0.0001 | 1.81 | 0.002 |
| *Slc16a6* | 1.73 | 0.0002 | – | – | 2.90 | <0.0001 |
| *Abcc3* | 1.70 | <0.0001 | – | – | 1.30 | 0.0002 |
| *Ddx3x* | 1.60 | <0.0001 | 0.33 | <0.0001 | 0.82 | 0.0007 |
| *Oaz1* | 1.53 | 0.0002 | – | – | 0.73 | 0.02 |
| *Me2* | 1.51 | 0.02 | – | – | 0.88 | 0.009 |
| *Soat1* | 1.44 | 0.01 | – | – | 0.96 | 0.03 |
| *Nampt* | 1.44 | 0.001 | – | – | 1.58 | 0.05 |
| *Snx8* | 1.43 | 0.02 | – | – | 0.96 | 0.005 |
| *Baz1a* | 1.39 | 0.002 | 0.66 | <0.0001 | 1.27 | 0.008 |
| *Pim1* | 1.38 | 0.0004 | – | – | 1.42 | <0.0001 |
| *Rgs1* | 1.35 | 0.002 | – | – | 2.47 | 0.04 |
| *Lhfpl2* | 1.27 | 0.006 | – | – | 1.50 | 0.02 |
| *Arpc5* | 1.24 | 0.01 | – | – | 0.59 | 0.02 |
| *Irak1* | 1.22 | 0.02 | – | – | 1.06 | 0.003 |
| *Glipr2* | 1.21 | 0.004 | – | – | 1.04 | 0.003 |
| *Hpse* | 1.20 | 0.02 | – | – | 2.24 | 0.008 |
| *Ada* | 1.17 | 0.03 | – | – | 0.68 | 0.03 |
| *Lims1* | 1.12 | 0.02 | – | – | 0.70 | 0.02 |
| *Il10rb* | 1.11 | 0.01 | – | – | 0.70 | 0.02 |
| *Sat1* | 1.09 | 0.01 | 0.33 | <0.0001 | 2.54 | 0.0002 |
| *Stx6* | 1.05 | 0.02 | – | – | 0.55 | 0.02 |
| *Klf4* | 1.04 | 0.008 | – | – | 1.08 | 0.01 |
| *Gng5* | 1.01 | 0.03 | – | – | 0.69 | 0.005 |
| *Map1b* | –1.00 | 0.01 | – | – | –1.00 | 0.04 |
| *Sestd1* | –1.05 | 0.02 | – | – | –1.18 | 0.02 |
| *Dip2c* | –1.05 | 0.01 | – | – | –1.15 | 0.02 |
| *Lipt1* | –1.10 | 0.04 | – | – | –1.18 | 0.002 |
| *Tnrc6b* | –1.12 | 0.004 | – | – | –0.56 | 0.03 |
| *Slc6a1* | –1.12 | 0.03 | – | – | –2.40 | 0.0006 |
| *Thyn1* | –1.13 | 0.03 | – | – | –1.40 | 0.004 |
| *Mrps9* | –1.14 | 0.008 | – | – | –1.06 | 0.04 |
| *Trim36* | –1.14 | 0.02 | – | – | –2.18 | 0.002 |
| *Rab33b* | –1.15 | 0.02 | – | – | –1.22 | 0.01 |
| *Cep68* | –1.15 | 0.01 | – | – | –1.12 | 0.0002 |
| *Mpp7* | –1.21 | 0.006 | 2.56 | <0.0001 | –1.18 | 0.001 |
| *Trdmt1* | –1.23 | 0.008 | – | – | –0.49 | 0.05 |
| *Aldh3a2* | –1.25 | 0.01 | – | – | –0.51 | 0.04 |
| *Ints2* | –1.26 | 0.001 | – | – | –0.79 | 0.02 |
| *Slc25a12* | –1.28 | 0.002 | – | – | –1.32 | 0.01 |
| *Ankmy2* | –1.31 | 0.03 | – | – | –1.25 | 0.02 |
| *Kank1* | –1.35 | 0.0003 | – | – | –1.89 | 0.05 |
| *Actr6* | –1.38 | 0.003 | – | – | –1.12 | 0.05 |
| *Tsn* | –1.42 | 0.001 | – | – | –0.58 | 0.02 |
| *Mettl14* | –1.44 | 0.007 | – | – | –0.86 | 0.03 |
| *Synj2bp* | –1.46 | 0.001 | – | – | –0.92 | 0.05 |
| *Pogz* | –1.46 | 0.0002 | – | – | –0.74 | 0.008 |
| *Ccdc102a* | –1.48 | 0.0003 | – | – | –0.62 | 0.03 |
| *Pin4* | –1.50 | 0.004 | – | – | –0.54 | 0.05 |
| *Parp2* | –1.53 | 0.0007 | – | – | –1.29 | 0.0003 |
| *Akap1* | –1.57 | <0.0001 | – | – | –1.00 | 0.006 |
| *Etaa1* | –1.59 | 0.0004 | – | – | –0.84 | 0.003 |
| *Sin3a* | –1.59 | <0.0001 | – | – | –0.74 | 0.02 |
| *Armc1* | –1.59 | 0.0005 | – | – | –0.58 | 0.02 |
| *Cbr3* | –1.61 | 0.006 | – | – | –1.18 | 0.005 |
| *Svil* | –1.62 | <0.0001 | – | – | –0.92 | 0.04 |
| *Ndn* | –1.64 | 0.0003 | – | – | –1.22 | 0.03 |
| *Ttc32* | –1.68 | 0.001 | – | – | –1.18 | 0.009 |
| *Dut* | –1.70 | 0.0001 | – | – | –0.49 | 0.02 |
| *Gucy1b3* | –1.72 | 0.002 | – | – | –1.12 | 0.003 |
| *Rfc1* | –1.75 | <0.0001 | – | – | –0.79 | 0.03 |
| *Lmo7* | –1.81 | <0.0001 | – | – | –1.00 | 0.04 |
| *Fabp3* | –1.81 | <0.0001 | – | – | –1.09 | 0.03 |
| *Unc13b* | –1.82 | <0.0001 | – | – | –1.00 | 0.04 |
| *Klhdc2* | –1.85 | <0.0001 | – | – | –1.12 | 0.03 |
| *Efhd1* | –1.89 | 0.0004 | – | – | –2.12 | 0.03 |
| *Habp4* | –1.91 | <0.0001 | – | – | –0.97 | 0.002 |
| *Sass6* | –1.96 | <0.0001 | – | – | –0.67 | 0.05 |
| *Spata18* | –1.96 | 0.0002 | – | – | –0.74 | 0.01 |
| *Eef1a1* | –2.04 | 0.0002 | – | – | –1.22 | 0.03 |
| *Abhd10* | –2.14 | <0.0001 | – | – | –0.62 | 0.02 |
| *Afap1l2* | –2.19 | <0.0001 | – | – | –0.60 | 0.04 |
| *Mylip* | –2.41 | <0.0001 | – | – | –1.89 | 0.03 |
| *Casd1* | –2.67 | <0.0001 | – | – | –1.09 | 0.02 |
| *Hey2* | –2.67 | <0.0001 | – | – | –2.56 | <0.0001 |
| *Hist1h4c* | –3.20 | <0.0001 | – | – | –0.81 | 0.03 |
| *Lrrn3* | – | – | –0.71 | <0.0001 | –0.97 | 0.004 |
| *Itpr1* | – | – | –0.65 | <0.0001 | –1.64 | <0.0001 |
| *Trib2* | – | – | –0.56 | <0.0001 | –0.86 | 0.03 |
| *Csrp2* | – | – | –0.47 | <0.0001 | –2.32 | 0.02 |
| *Nrn1* | – | – | –0.47 | <0.0001 | –2.25 | 0.0006 |
| *Id3* | – | – | –0.42 | <0.0001 | –1.43 | 0.04 |
| *Obsl1* | – | – | –0.36 | <0.0001 | –0.64 | 0.01 |
| *Camk2g* | – | – | –0.30 | <0.0001 | –1.32 | <0.0001 |
| *Isyna1* | – | – | –0.25 | <0.0001 | –0.84 | 0.01 |
| *Wipi1* | – | – | 0.26 | <0.0001 | 0.87 | 0.006 |
| *Pla2g15* | – | – | 0.28 | <0.0001 | 0.75 | 0.04 |
| *Top1* | – | – | 0.28 | <0.0001 | 1.12 | 0.0007 |
| *Bcat1* | – | – | 0.29 | <0.0001 | 1.91 | 0.01 |
| *Cklf* | – | – | 0.29 | <0.0001 | 1.15 | 0.003 |
| *Slc16a3* | – | – | 0.29 | <0.0001 | 0.78 | 0.03 |
| *Glrx* | – | – | 0.30 | <0.0001 | 1.09 | 0.002 |
| *Sra1* | – | – | 0.31 | <0.0001 | 0.79 | 0.01 |
| *S100a11* | – | – | 0.33 | <0.0001 | 0.98 | 0.03 |
| *Tpi1* | – | – | 0.34 | <0.0001 | 0.72 | 0.03 |
| *Papss2* | – | – | 0.39 | <0.0001 | 1.23 | 0.05 |
| *Gas7* | – | – | 0.40 | <0.0001 | 0.78 | 0.03 |
| *Ptpre* | – | – | 0.41 | <0.0001 | 1.37 | 0.03 |
| *Sphk1* | – | – | 0.70 | <0.0001 | 1.98 | 0.002 |
| *Tbc1d8* | – | – | 0.72 | <0.0001 | 0.97 | 0.03 |
| *Psap* | – | – | 0.73 | <0.0001 | 0.77 | 0.05 |
| *Ostf1* | – | – | 0.77 | <0.0001 | 1.63 | <0.0001 |
| *Cxcr4* | – | – | 0.92 | <0.0001 | 2.33 | 0.01 |
| *Cebpb* | – | – | 1.04 | <0.0001 | 1.24 | 0.02 |
| *S100a10* | – | – | 1.20 | <0.0001 | 1.24 | 0.002 |
| *Lyve1* | – | – | 1.28 | <0.0001 | 0.92 | 0.04 |

FC, fold change.

Liu *et al*. (2017) and Kurki *et al*. (2011) denoted genes as upregulated in ruptured tissue relative to unruptured tissue. Park *et al*. (2020) used the opposite convention, with genes denoted as upregulated in unruptured relative to ruptured tissue. We therefore have transformed the results of Park *et al*. (2020) to reflect the convention used in the other studies for ease of comparison.
