# Supplementary material for: Common mechanisms of physiological and pathological rupture events in biology: novel insights into mammalian ovulation and beyond
Source: Biol Rev Camb Philos Soc. Author manuscript; Available in PMC 2023 Oct 1. (PMC10524764; doi:10.1111/brv.12970)
Supplement: Table S3 — Complete list of overlapping upregulated and downregulated genes in comparisons between the intracranial aneurysm (ICA) and chorioamniotic membrane rupture (CMR) data sets. [file NIHMS1902325-supplement-Table_S3.docx]

**Table S3.** Complete list of overlapping upregulated (+) and downregulated (–) genes in comparisons between the intracranial aneurysm (ICA; Kurki *et al*., 2011) and chorioamniotic membrane rupture (CMR; Nhan-Chang *et al*., 2010) data sets.

| **Gene** | **ICA Log2FC** | **ICA**  **P-value** | **CMR Log2FC** | **CMR**  **P-value** |
| --- | --- | --- | --- | --- |
| *C15orf48* | 4.44 | 0.005 | 1.8 | 0.009 |
| *IL8* | 3.31 | 0.04 | 2 | 0.006 |
| *UPP1* | 2.57 | 0.04 | 1.6 | 0.03 |
| *CCL20* | 2.33 | 0.0004 | 2.6 | 0.0008 |
| *PLAUR* | 2.23 | 0.02 | 1.5 | 0.0002 |
| *COL11A1* | 2.19 | 0.03 | 2.6 | 0.0003 |
| *IL6* | 2.18 | 0.03 | 2.7 | 0.0004 |
| *TNFAIP3* | 2.09 | 0.007 | 1.5 | 0.01 |
| *GNA15* | 1.75 | 0.04 | 1.7 | 0.005 |
| *IER3* | 1.73 | 0.0001 | 2.1 | 0.01 |
| *MT2A* | 1.60 | 0.02 | 1.6 | 0.04 |
| *NAMPT* | 1.59 | 0.0009 | 1.6 | 0.003 |
| *DUSP5* | 1.56 | 0.005 | 1.7 | 0.003 |
| *TMEM158* | 1.38 | 0.01 | 2 | 0.0002 |
| *SLC7A5* | 1.21 | 0.03 | 1.9 | 0.06 |
| *TSPAN13* | 1.19 | 0.01 | 1.7 | 0.0007 |
| *CA2* | 1.15 | 0.03 | 1.7 | 0.0008 |
| *TGFBI* | 1.14 | 0.03 | 1.6 | 0.0007 |
| *SLC25A37* | 1.02 | 0.005 | 1.6 | 0.0007 |
| *SLCO4A1* | 0.87 | 0.03 | 1.6 | 0.0007 |
| *C12orf59* | 0.86 | 0.02 | 1.5 | 0.01 |
| *C3orf52* | 0.82 | 0.01 | 1.6 | 0.0006 |
| *NUAK2* | 0.71 | 0.01 | 1.9 | 0.09 |
| *PTGES* | 0.64 | <0.0001 | 1.6 | 0.03 |
| *SERINC2* | 0.51 | 0.009 | 1.7 | 0.001 |
| *ACACB* | –0.62 | 0.007 | –1.6 | 0.0008 |
| *DYNC2LI1* | –0.62 | 0.004 | –1.6 | 0.04 |
| *SNX21* | –0.62 | 0.02 | –1.6 | 0.008 |
| *PGM5* | –0.64 | 0.003 | –3.3 | 0.001 |
| *SNCAIP* | –0.64 | 0.002 | –1.9 | 0.0004 |
| *C21orf34* | –0.69 | 0.04 | –1.8 | 0.001 |
| *PCCA* | –0.74 | 0.02 | –2.1 | 0.01 |
| *DTNA* | –0.81 | 0.02 | –1.6 | 0.009 |
| *NFIB* | –1.12 | 0.03 | –2 | 0.07 |
| *ZBTB20* | –1.12 | 0.03 | –1.5 | 0.002 |
| *MFAP4* | –1.18 | 0.02 | –2.3 | 0.08 |
| *PDGFRB* | –1.29 | 0.01 | –1.7 | 0.06 |
| *PLCL1* | –1.36 | 0.04 | –1.8 | 0.03 |
| *SRPX* | –1.36 | 0.03 | –1.8 | 0.07 |
| *C21orf63* | –1.40 | 0.0005 | –1.9 | 0.005 |
| *GSTA4* | –1.43 | 0.02 | –1.6 | 0.002 |
| *PCDH18* | –1.43 | 0.02 | –1.7 | 0.0007 |
| *MAP3K5* | –1.60 | 0.02 | –1.9 | 0.06 |
| *HEPH* | –1.64 | 0.05 | –1.7 | 0.02 |
| *EBF1* | –1.69 | <0.0001 | –1.8 | 0.01 |
| *GJA4* | –1.74 | 0.003 | –1.6 | 0.01 |
| *FBXL2* | –1.79 | 0.005 | –1.5 | 0.06 |
| *MAMDC2* | –1.84 | 0.03 | –2.4 | 0.002 |
| *PLSCR4* | –1.84 | 0.005 | –1.7 | 0.005 |
| *NCALD* | –1.94 | 0.002 | –1.5 | 0.0004 |
| *RAMP1* | –1.94 | 0.04 | –2.2 | 0.0006 |
| *NR2F2* | –2.06 | 0.02 | –1.7 | 0.001 |
| *DKFZP564O0823* | –2.12 | 0.002 | –3 | 0.0005 |
| *ENPP2* | –2.18 | 0.01 | –3.7 | 0.0008 |
| *MYH11* | –2.25 | 0.006 | –2.7 | 0.09 |
| *RGS5* | –2.32 | 0.01 | –2 | 0.02 |
| *NAP1L3* | –2.40 | 0.05 | –2.2 | 0.0008 |
| *EMX2* | –2.64 | 0.0006 | –1.7 | 0.0002 |
| *PPP1R14A* | –2.64 | 0.006 | –1.6 | 0.002 |
| *ECM2* | –3.32 | 0.009 | –1.6 | 0.03 |

FC, fold change.

Note: upregulated genes are upregulated in ruptured tissue.
