# Supplementary material for: Common mechanisms of physiological and pathological rupture events in biology: novel insights into mammalian ovulation and beyond
Source: Biol Rev Camb Philos Soc. Author manuscript; Available in PMC 2023 Oct 1. (PMC10524764; doi:10.1111/brv.12970)
Supplement: Table S2 — Complete list of overlapping upregulated and downregulated genes in comparisons between two ovulation data sets and one data set for chorioamniotic membrane rupture (CMR). [file NIHMS1902325-supplement-Table_S2.docx]

**Table S2.** Complete list of overlapping upregulated and downregulated genes in comparisons between two ovulation data sets and one data set for chorioamniotic membrane rupture (CMR).

| **Gene** | **Ovulation (Liu *et al*., 2017) Log2FC** | **Ovulation (Liu *et al*., 2017)**  **P-value** | **Ovulation (Park *et al*., 2020)**  **Log2FC** | **Ovulation (Park *et al*., 2020)**  **P-value** | **CMR (Nhan-Chang *et al*., 2010) Log2FC** | **CMR (Nhan-Chang *et al*., 2010)**  **P-value** |
| --- | --- | --- | --- | --- | --- | --- |
| Rhpn2 | 5.00 | <0.0001 | – | – | 1.60 | 0.02 |
| Plekhg6 | 3.52 | <0.0001 | – | – | 1.50 | 0.02 |
| Ptgs2 | 3.25 | <0.0001 | – | – | 2.10 | 0.005 |
| Uck2 | 2.99 | <0.0001 | – | – | 1.60 | 0.006 |
| Plod2 | 2.80 | <0.0001 | – | – | 1.80 | 0.0008 |
| Net1 | 2.79 | <0.0001 | – | – | 1.90 | 0.0008 |
| Slc7a5 | 2.69 | <0.0001 | – | – | 1.90 | 0.0004 |
| Pfkfb4 | 2.60 | <0.0001 | 0.45 | <0.0001 | 1.50 | 0.009 |
| Nedd9 | 2.44 | <0.0001 | – | – | 1.70 | 0.02 |
| Nt5e | 2.37 | <0.0001 | – | – | 1.50 | 0.004 |
| Dsg2 | 2.21 | <0.0001 | – | – | 1.60 | 0.01 |
| F2rl1 | 2.06 | <0.0001 | – | – | 1.60 | 0.002 |
| Pdgfb | 1.97 | <0.0001 | – | – | 1.50 | 0.05 |
| Krt18 | 1.93 | <0.0001 | – | – | 1.70 | 0.008 |
| Lox | 1.92 | 0.0002 | 0.28 | 0.005 | 2.30 | 0.001 |
| Aqp1 | 1.88 | 0.0002 | – | – | 1.70 | 0.008 |
| Phlda2 | 1.84 | <0.0001 | – | – | 1.60 | 0.02 |
| Ppp1r13l | 1.83 | <0.0001 | – | – | 1.50 | 0.005 |
| Angptl4 | 1.79 | <0.0001 | 0.32 | <0.0001 | 2.40 | 0.0009 |
| Epha2 | 1.64 | 0.003 | – | – | 1.60 | 0.02 |
| Tnip1 | 1.56 | 0.0009 | – | – | 1.50 | 0.02 |
| Fstl1 | 1.50 | 0.003 | – | – | 1.60 | 0.02 |
| Nampt | 1.43 | 0.001 | – | – | 1.60 | 0.03 |
| Flnc | 1.41 | 0.02 | – | – | 2.00 | 0.008 |
| Col5a1 | 1.37 | 0.02 | – | – | 1.60 | 0.003 |
| Atp1b1 | 1.32 | 0.002 | – | – | 1.60 | 0.003 |
| Fosl1 | 1.28 | 0.002 | – | – | 1.70 | 0.004 |
| Spint2 | 1.23 | 0.002 | – | – | 1.70 | 0.01 |
| Ahnak | 1.17 | 0.03 | – | – | 1.60 | 0.08 |
| Spry4 | 1.10 | 0.005 | – | – | 1.50 | 0.02 |
| Nav3 | –1.05 | 0.005 | – | – | –1.80 | 0.001 |
| Slc31a2 | –1.06 | 0.02 | – | – | –1.80 | 0.02 |
| Scpep1 | –1.13 | 0.006 | – | – | –1.70 | 0.02 |
| Stra6 | –1.34 | 0.002 | – | – | –1.60 | 0.05 |
| Lpar1 | –1.42 | 0.001 | – | – | –1.70 | 0.03 |
| Pcbd1 | –1.48 | 0.007 | – | – | –1.50 | 0.03 |
| Mov10l1 | –1.52 | 0.0007 | – | – | –1.50 | 0.0009 |
| Ldhb | –1.67 | 0.0006 | – | – | –1.60 | 0.003 |
| Fhl1 | –1.74 | 0.0005 | – | – | –1.60 | 0.07 |
| Cst3 | –1.81 | 0.0004 | – | – | –1.50 | 0.0009 |
| Ung | –1.82 | <0.0001 | – | – | –1.60 | 0.0002 |
| Vcan | –2.09 | 0.001 | – | – | –1.60 | 0.08 |
| Zcchc24 | –2.13 | <0.0001 | – | – | –1.80 | 0.001 |
| Apod | –2.25 | <0.0001 | – | – | –3.60 | 0.05 |
| Chek2 | –2.31 | <0.0001 | – | – | –1.60 | 0.0002 |
| Cyp1b1 | –2.40 | <0.0001 | – | – | –2.10 | 0.0008 |
| Acp5 | –2.41 | <0.0001 | – | – | –1.70 | 0.004 |
| A2m | –3.31 | <0.0001 | – | – | –2.10 | 0.01 |
| Avpi1 | – | – | –0.54 | <0.0001 | –1.60 | 0.006 |
| St6gal1 | – | – | –0.50 | <0.0001 | –1.60 | 0.001 |
| Tox2 | – | – | –0.39 | <0.0001 | –1.70 | 0.004 |
| Rprm | – | – | –0.37 | <0.0001 | –1.90 | 0.02 |
| Mgp | – | – | –0.28 | <0.0001 | –2.60 | 0.007 |
| Col18a1 | – | – | –0.28 | <0.0001 | –1.60 | 0.09 |
| Itm2a | – | – | –0.26 | <0.0001 | –2.30 | 0.0003 |
| Aldh1a1 | – | – | –0.26 | <0.0001 | –1.90 | 0.07 |
| St5 | – | – | 0.26 | <0.0001 | 1.60 | 0.001 |
| Cdh2 | – | – | 0.30 | <0.0001 | 1.70 | 0.02 |
| Scg2 | – | – | 0.32 | <0.0001 | 1.50 | 0.04 |
| Pthlh | – | – | 0.36 | <0.0001 | 2.50 | 0.004 |
| Rnf217 | – | – | 0.46 | <0.0001 | 1.60 | 0.005 |
| Comp | – | – | 0.51 | <0.0001 | 3.60 | 0.0008 |
| Stxbp6 | – | – | 1.11 | <0.0001 | 1.80 | 0.006 |

FC, fold change.

Liu *et al*. (2017) and Nhan-Chang *et al*. (2010) denoted genes as upregulated in ruptured tissue relative to unruptured tissue. Park *et al*. (2020) used the opposite convention, with genes denoted as upregulated in unruptured relative to ruptured tissue. We therefore have transformed the results of Park *et al*. (2020) to reflect the convention used in the other studies for ease of comparison.
